# Ferromagnetic-antiferromagnetic coexisting ground state and exchange bias effects in MnBi_4_Te_7_ and MnBi_6_Te_10_

**DOI:** 10.1038/s41467-022-35184-7

**Published:** 2022-12-10

**Authors:** Xiaolong Xu, Shiqi Yang, Huan Wang, Roger Guzman, Yuchen Gao, Yaozheng Zhu, Yuxuan Peng, Zhihao Zang, Ming Xi, Shangjie Tian, Yanping Li, Hechang Lei, Zhaochu Luo, Jinbo Yang, Yeliang Wang, Tianlong Xia, Wu Zhou, Yuan Huang, Yu Ye

**Affiliations:** 1 State Key Laboratory for Mesoscopic Physics and Frontiers Science Center for Nano-optoelectronics, School of Physics, Peking University, Beijing, 100871 China; 2 School of Integrated Circuits and Electronics, MIIT Key Laboratory for Low-Dimensional Quantum Structure and Devices, Beijing Institute of Technology, Beijing, 100081 China; 3 Collaborative Innovation Center of Quantum Matter, Beijing, 100871 China; 4 Academy for Advanced Interdisciplinary Studies, Peking University, Beijing, 100871 China; 5 Beijing Key Laboratory of Opto-Electronic Functional Materials & Micro-Nano Devices, Department of Physics, Renmin University of China, Beijing, 100872 China; 6 School of Physical Sciences, University of Chinese Academy of Sciences, Beijing, 100049 China; 7 Laboratory for Neutron Scattering, Department of Physics, Renmin University of China, Beijing, 100872 China; 8 CAS Centre for Excellence in Topological Quantum Computation, University of Chinese Academy of Sciences, Beijing, 100049 China; 9 Advanced Research Institute of Multidisciplinary Science, Beijing Institute of Technology, Beijing, 100081 China; 10 Yangtze Delta Institute of Optoelectronics, Peking University, Nantong, 226010 Jiangsu China

**Keywords:** Two-dimensional materials, Magnetic properties and materials

## Abstract

Natural superlattice structures MnBi_2_Te_4_(Bi_2_Te_3_)_*n*_ (*n* = 1, 2, ...), in which magnetic MnBi_2_Te_4_ layers are separated by nonmagnetic Bi_2_Te_3_ layers, hold band topology, magnetism and reduced interlayer coupling, providing a promising platform for the realization of exotic topological quantum states. However, their magnetism in the two-dimensional limit, which is crucial for further exploration of quantum phenomena, remains elusive. Here, complex ferromagnetic-antiferromagnetic coexisting ground states that persist down to the 2-septuple layers limit are observed and comprehensively investigated in MnBi_4_Te_7_ (*n* = 1) and MnBi_6_Te_10_ (*n* = 2). The ubiquitous Mn-Bi site mixing modifies or even changes the sign of the subtle interlayer magnetic interactions, yielding a spatially inhomogeneous interlayer coupling. Further, a tunable exchange bias effect, arising from the coupling between the ferromagnetic and antiferromagnetic components in the ground state, is observed in MnBi_2_Te_4_(Bi_2_Te_3_)_*n*_ (*n* = 1, 2), which provides design principles and material platforms for future spintronic devices. Our work highlights a new approach toward the fine-tuning of magnetism and paves the way for further study of quantum phenomena in MnBi_2_Te_4_(Bi_2_Te_3_)_*n*_ (*n* = 1, 2) as well as their magnetic applications.

## Abstract (web summary)

MnBi_2_Te_4_ and Bi_2_Te_3_ can form natural superlattices, where the MnBi_2_Te_4_ layers are separated by multiples of Bi_2_Te_3_. The combination of these two materials offers a potential platform for the interplay of tunable magnetism and topology. Here, the authors show that MnBi_4_Te_7_ and MnBi_6_Te_10_ display a complex magnetic ground state with coexisting ferromagnetic and antiferromagnetic domains.

## Introduction

The interplay between magnetism and topology inaugurates a new horizon in exploring exotic quantum phenomena, such as the quantum anomalous Hall effect (QAHE), axion insulators and magnetic Weyl semimetals^1–6^. Recently, MnBi_2_Te_4_ (MBT) was discovered to be an intrinsic stoichiometric antiferromagnetic (AFM) topological insulator (TI)^7–14^. The intertwined band topology and magnetic order in A-type AFM MnBi_2_Te_4_ pose great challenges to the realization of its exotic topological physics and subsequent devices, because either the layer number of the material needs to be strictly controlled, or a high magnetic field (~6–8 T) is required^11–14^. Therefore, there is an urgent need to develop a magnetic topological insulator of the MBT family that is magnetically insensitive to the number of layers and has a small saturation field, in which engineering of the interlayer magnetic interaction is the key. Now, the natural superlattice structure MnBi_2_Te_4_(Bi_2_Te_3_)_*n*_ (*n* = 1, 2, ...) provides an opportunity to modify the interlayer exchange coupling using nonmagnetic Bi_2_Te_3_ (BT) quintuple layer (QL) as spacer layers^15–20^. With the increase of *n*, the interlayer AFM coupling gradually weakens, while the global spin-orbit coupling strength gradually increases with the increase of Bi content^16–21^. For MnBi_4_Te_7_ (*n* = 1) and MnBi_6_Te_10_ (*n* = 2), neutron scattering experiments and theoretical calculations show that they possess weak interlayer magnetic coupling, but still maintain the A-type AFM structure^19–23^, that is, in each MBT septuple layer (SL), the Mn magnetic moments are ferromagnetically aligned, while the adjacent SLs are antiferromagnetically aligned. Although tremendous efforts have been devoted to studying the magnetic properties of the MnBi_4_Te_7_ and MnBi_6_Te_10_, most studies have only focused on their bulk phase^16–20,24–27^. However, the magnetic property and topological phase in MBT family often exhibit intricate thickness dependence, posing a significant influence for the realization of exotic topological physics^11–14,28–30^. Therefore, determining the magnetism of MnBi_2_Te_4_(Bi_2_Te_3_)_*n*_ (*n* = 1, 2) at their two-dimensional (2D) limit is crucial, but has so far remained elusive.

In this work, we systematically investigate the magnetic properties of MnBi_4_Te_7_ and MnBi_6_Te_10_ thin flakes down to 1 SL by employing polar reflective magnetic circular dichroism (RMCD) spectroscopy. We demonstrate that the odd-even-layer oscillation of compensated and uncompensated AFM states, a common effect in ideal A-type AFM materials^13,30–32^, vanishes in atomically thin MnBi_4_Te_7_ and MnBi_6_Te_10_. In all the measured samples above 1 SL, in addition to the AFM spin-flip transition, a significant ferromagnetic (FM) hysteresis loop centered at zero field is observed. We reveal that the peculiar hysteresis loop arises from the complex FM-AFM coexisting ground state. Atomic-resolution electron energy loss spectroscopy (EELS) mapping as well as single-crystal X-ray diffraction (SC-XRD) reveal the ubiquitous Mn-Bi site mixing in the crystals. The spins of the randomly distributed Mn_Bi_ antisite defects in each SL are antiferromagnetically coupled to the spins of the main Mn layer^23,24,33^, that further modify or even change the sign of the subtle inter-SL magnetic interactions, yielding a spatially inhomogeneous interlayer coupling. As a result, the energy gained through the formation of magnetic domains compensates for the energy cost in the appearance of domain walls, yielding a complex FM-AFM coexisting ground state. Furthermore, a tunable exchange bias effect is observed in MnBi_4_Te_7_ and MnBi_6_Te_10_, arising from the coupling between the FM and AFM components in the ground state. The direction of this exchange bias can be easily tuned by the historical polarization field and does not require warming and field cooling processes. Our results reveal the nontrivial magnetic ground states in MnBi_4_Te_7_ and MnBi_6_Te_10_, keeping the promise for further investigation of exotic topological quantum states.

## Results

### FM-AFM coexisting ground states of MnBi_4_Te_7_ and MnBi_6_Te_10_

High-quality MnBi_4_Te_7_ and MnBi_6_Te_10_ bulk crystals are grown by flux method^34^ and confirmed by XRD results (Supplementary Fig. S1). The field-cooled (FC) and zero-field-cooled (ZFC) magnetic susceptibilities of *H*∥*c* (*χ*^*c*^) of MnBi_4_Te_7_ and MnBi_6_Te_10_ crystals show that their long-range AFM orders occur at Néel temperature (*T*_N_) of 12.1 K and 10.9 K, respectively (Supplementary Fig. S2). Compared with MnBi_2_Te_4_^7,22^ (*T*_N_ of ~24.5 K), the reduction of *T*_N_ in MnBi_4_Te_7_ and MnBi_6_Te_10_ manifests the weakened interlayer coupling. Scrutiny of the *M*–*H* curve at 2 K under *H*∥*c*, we find that MnBi_4_Te_7_ (MnBi_6_Te_10_) undergoes a spin transition at a very low magnetic field and quickly enters the forced FM state with a field of about 0.25 T (0.21 T). This is in sharp contrast to MnBi_2_Te_4_, where the spin-flop transition occurs at about 3.5 T and its magnetic moment eventually saturates under external magnetic fields larger than 8 T^11–13,30^ (Supplementary Fig. S3a). We can estimate the interlayer antiferromagnetic coupling *J*_c_ and single-ion anisotropy *D* based on the Stoner–Wohlfarth model^35^, giving *SJ*_c_ ≈ 0.0127 meV and *SD* ≈ 0.0440 meV in MnBi_4_Te_7_ and *SJ*_c_ ≈ 0.0037 meV and *SD* ≈ 0.0417 meV in MnBi_6_Te_10_ (see details in Supplementary Note I). The anisotropy energy of MnBi_4_Te_7_ and MnBi_6_Te_10_ are of the same order of magnitudes as that of MnBi_2_Te_4_, but the interlayer coupling values of MnBi_4_Te_7_ and MnBi_6_Te_10_ are almost 1–2 orders of magnitude smaller than that of MnBi_2_Te_4_^19,30,33,36^, indicating a greatly reduced interlayer coupling from MnBi_2_Te_4_ to MnBi_4_Te_7_ and MnBi_6_Te_10_.

**Fig. 1:**
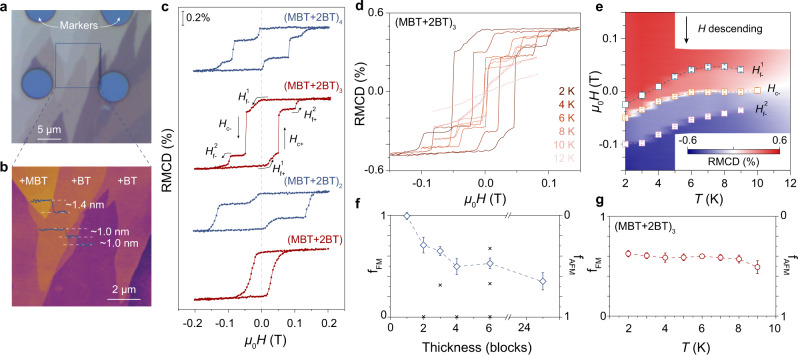
FM-AFM coexisting magnetic ground state in MnBi_6_Te_10_. **a** A typical optical image of the exfoliated MnBi_6_Te_10_ sample with different thicknesses and terminations. **b** Atomic force microscopy height image and height line profiles measured in the area marked by the black box in (**a**), showing a thickness of ~1.4 nm for MnBi_2_Te_4_ SL and ~1.0 nm for Bi_2_Te_3_ QL. **c** RMCD signals versus *μ*_0_*H* for 1 to 4-SL (terminated by 2-QL protection layer) MnBi_6_Te_10_ flakes at 2 K. The subscripts “+” and “−” denote the field-sweeping direction, and superscripts “1” and “2” denote the first and second AFM transitions. **d** RMCD sweeps for the 3-SL MnBi_6_Te_10_ flake at a temperature range that passes through its *T*_N_. **e** 2D plot of RMCD signal as functions of temperature and magnetic field (descending sweep) for the 3-SL MnBi_6_Te_10_ flake. Two AFM spin-flip fields (${H}_{{{{{{{{\rm{f-}}}}}}}}}^{1}$ and ${H}_{{{{{{{{\rm{f-}}}}}}}}}^{2}$) and one FM spin-flip field (*H*_c–_) are extracted and superimposed on the plot. The error bars correspond to the range of the transition during each spin flip. **f**, **g** Proportions of FM (*f*_FM_, left axis) and AFM (*f*_AFM_, right axis) component as functions of thickness (**f**) and temperature (**g**). Cross marks in (**f**) indicate the expected values for the single-domain case. The vertical error bars are estimated from the experimental uncertainty of the measured signal.

To explore how the weakened interlayer exchange coupling affects the magnetic ground order, we investigated the layer-number-dependent magnetism of MnBi_4_Te_7_ and MnBi_6_Te_10_ using the RMCD spectroscopy. Atomically thin flakes down to 1 SL are mechanically exfoliated from their bulk crystals onto gold substrates using the standard Scotch tape method^37^ (Fig. 1a). The number of layers is confirmed by atomic force microscopy characterizations (see Supplementary Fig. S4 for MnBi_4_Te_7_ samples and Fig. S5 for MnBi_6_Te_10_ samples). Typical height line profiles of the stepped MnBi_6_Te_10_ flakes indicate SL and QL thicknesses of approximately 1.4 and 1.0 nm, respectively (Fig. 1b), consistent with previous reports^29,38^. The layer-number-dependent magnetic behavior in MnBi_4_Te_7_ and MnBi_6_Te_10_ is very similar, so here we show 1–4 SLs MnBi_6_Te_10_ as examples, and provide the full set of other samples in Supplementary Figs. S6 and S7. 1-SL MnBi_6_Te_10_ shows a distinct RMCD signal at zero field and a clear hysteresis loop (Fig. 1c), confirming its FM nature. With increasing temperature, the hysteresis loop shrinks and disappears at 10 K, indicating an FM to paramagnetic phase transition (Supplementary Fig. S8a). Compared with the intrinsic 1-SL MnBi_2_Te_4_ (*T*_C_ = 14.5 K)^30^, the decreased *T*_C_ may be due to the increased Bi_Mn_ sites, as the intralayer exchange coupling decreases with the increase of the average distance between the Mn sites. Surprisingly, with increasing thickness, no odd-even layer-number oscillation occurs, which is a typical feature of ideal A-type AFM materials. Instead, both FM and AFM signals (confirmed by the following discussions) are present in all the measured samples with SL number *N* > 1 (Fig. 1c). In the 2-SL sample, there is a distinct residual magnetic moment after spin-flipping near zero field during the descending field, indicating the existence of additional FM component. Here we denote the transition fields of the AFM and FM components as *H*_f_ and *H*_c_, respectively. With increasing thickness, the magnetic reversal curve evolves into two AFM and one FM spin-flip transitions with different magnetic moment amplitudes (Fig. 1c).

As the temperature increases, the value of the spin-flip field ${H}_{{{{{{{{\rm{f-}}}}}}}}}^{1}$ in the 3-SL sample changes from negative to positive, and then slowly decreases to approach zero (Fig. 1d, e). A positive value of ${H}_{{{{{{{{\rm{f-}}}}}}}}}^{1}$ again signifies the AFM nature of this spin-flip process, since interlayer AFM coupling prefers to make the magnetic moment in adjacent layers be antiparallel, while the Zeeman energy tends to keep the magnetic moment parallel to the magnetic field. Since the AFM interlayer coupling is stronger in the MnBi_4_Te_7_ samples, ${H}_{{{{{{{{\rm{f-}}}}}}}}}^{1}$ usually occurs at larger positive values (see Supplementary Figs. S6 and S9). The FM spin-flip field *H*_c–_ of the MnBi_6_Te_10_ samples increase monotonically from a negative value and approach zero with increasing temperature (Fig. 1d, e and Supplementary Fig. S8b). However, in some MnBi_4_Te_7_ samples (Supplementary Fig. S9d), *H*_c–_ first increases from negative to positive and then jumps back to negative with increasing temperature, signifying the correlated coupling between the FM and AFM components.

Specifically, we build a five-layer macrospin model to interpret the hysteresis loops in the MnBi_4_Te_7_ and MnBi_6_Te_10_ samples (Supplementary Fig. S10), which captures the main features. But in fact, the FM and AFM distributions and proportions can be very complex due to the spatial inhomogeneity. We denote the proportions of FM and AFM components by ${f}_{{{{{{{{\rm{FM}}}}}}}}}=\frac{{{\mbox{RMCD(FM flip height)}}}}{{{\mbox{RMCD(up-saturation)}}}-{{\mbox{RMCD(down-saturation)}}}}$ and *f*_AFM_ = 1 − *f*_FM_, respectively. As expected, *f*_AFM_ ~ 0 (*f*_FM_ ~ 1) in the 1-SL sample (Fig. 1f). The *f*_AFM_ (*f*_FM_) of MnBi_6_Te_10_ samples increases (decreases) as the number of layers increases. In most samples, the values of *f*_AFM_ do not coincide with those expected for single-domain case (cross marks in Fig. 1f), indicating a multi-domain configuration within the laser spot size. The values of *f*_FM_ and *f*_AFM_ are nearly temperature independent over the entire measurement temperature range (Fig. 1g).

After confirming the FM-AFM coexistence ground states, the puzzling magnetism of bulk MnBi_4_Te_7_ and MnBi_6_Te_10_ can also be well explained. It is worth noting that at low temperatures, the MnBi_4_Te_7_ and MnBi_6_Te_10_ crystals exhibit non-zero magnetization at zero field^8,16–19,22,29^, and the magnetic reversal completes through three sluggish spin-flip transitions (marked by the arrows in Supplementary Fig. S3). The non-zero magnetization at the zero field suggests that there may be some residual FM components in the AFM sate. The FM-AFM coexisting magnetic order is also confirmed by the bifurcations of the ZFC and FC curves at temperatures slightly below the Néel temperature (Supplementary Fig. S2). The large difference in the values of *f*_AFM_ between thin flakes and bulk crystals suggests that the FM-AFM coexisting magnetic order is possibly a thickness-related effect. The complex multi-domain structure in the bulk samples smears the distinct signatures of the FM-AFM coexisting ground state, resulting in sluggish changes in the magnetic moment, which also masks the potential applications of this unique magnetic order.

### Origin of the FM-AFM coexisting ground state

**Fig. 2:**
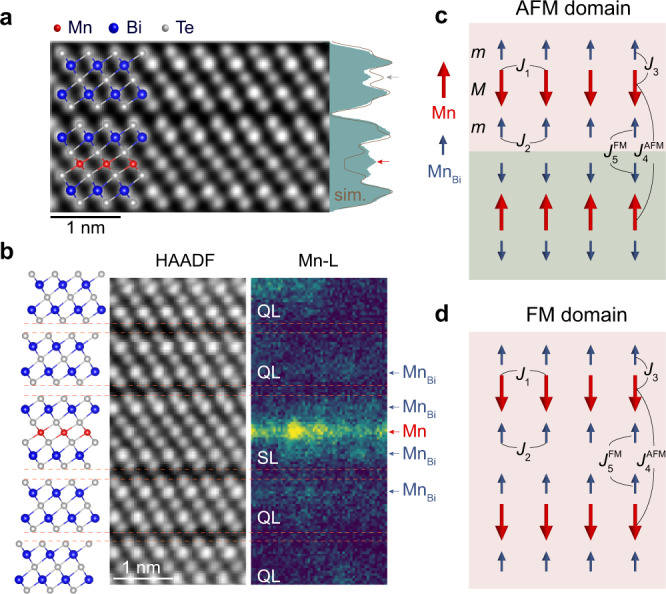
STEM characterizations and an intuitive model of inter-SL FM and AFM orders in MnBi_4_Te_7_ and MnBi_6_Te_10_. **a** Cross-sectional atomic-resolution HAADF image of the MnBi_6_Te_10_ crystal imaged along the [100] direction. Experimental (shaded area) and simulated (gray curve) integral HAADF intensity profiles along the *c*-axis show clear discrepancy, showing Bi_Mn_ antisite defects in SL (red arrow) and Mn_Te_ in QL (gray arrow). **b** Atomic structure and HAADF image of the MnBi_6_Te_10_ crystal with the corresponding EEELs mapping of the Mn element (L_2,3_ edge). Clear Mn signals are present in the Bi layers of SL and QL. The orange dashed lines indicate the van der Waals gaps. **c**, **d** Illustrations of the inter-SL FM and AFM regions in MnBi_4_Te_7_ and MnBi_6_Te_10_, in which the dominant magnetic exchange interactions are labeled.

Due to the weak interlayer coupling, the energy difference between the AFM and FM spin configurations is very small^19,20^, providing a breeding ground for the magnetism tuning^39,40^. Cross-sectional atomic-resolution high-angle annular dark-field scanning transmission electron microscopy (HAADF-STEM) image along the [100] direction shows that two Bi_2_Te_3_ layers are inserted between MnBi_2_Te_4_ layers in MnBi_6_Te_10_ (Fig. 2a, b). The crystal structure and stacking remain intact all the way to the surface, and no stacking disorders are observed (Supplementary Fig. S11). However, the experimental and simulated integrated HAADF intensity profiles along the *c*-axis show clear discrepancy, showing Bi_Mn_ antisite defects in SL (red arrow in Fig. 2a) and Mn_Te_ in QL (gray arrow in Fig. 2a). In addition, atomic EELS mapping shows clear Mn signals in the Bi layers in SL and also QL (Fig. 2b), demonstrating the existence of the Mn_Bi_ antisite defects (see Supplementary Fig. S11 for more details).

Below *T*_N_, the spins of Mn atoms are ferromagnetically coupled (−*J*_1_) within the SL, but antiferromagnetically coupled (*J*_4_) with those of Mn atoms in the adjacent SL. The spins of Mn_Bi_ in SL are ferromagnetically coupled (−*J*_2_) within the sublayer, but antiferromagnetically coupled (*J*_3_) to the main Mn layer^33^, forming a ferrimagnetic SL configuration (Fig. 2c). Flipping the spins of Mn_Bi_ in SL to align with those in the Mn layer requires a magnetic field above 50 T^33^, so all spins in SL flip together under a small magnetic field. This stable ferrimagnetic SL configuration results in a reduced measured saturation magnetic moment than the theoretically value^19^ (Supplementary Fig. S3), and determines that the magnetic moment change at low magnetic fields (from −0.2 to 0.2 T) comes only from that of the main Mn layer. Considering that Mn atoms doped into the Bi_2_Te_3_ always tend to be ferromagnetically coupled^41–43^, the spins of Mn_Bi_ are expected to be ferromagnetically coupled (−*J*_5_) in the adjacent SLs and within QLs. The magnetic moment in the QL to flip to align with the magnetic field requires a magnetic field of about 8 and 6 T in MnBi_4_Te_7_ and MnBi_6_Te_10_^24^, respectively (marked by the arrows in Supplementary Fig. S3). For simplification, we do not additionally consider the coupling in QL and accommodate it in *J*_5_, which effectively strengthens the FM coupling between the Mn_Bi_ antisites in the two neighboring SLs. Then, the Hamiltonian of the 2-SL system can be expressed as:

1 $$\begin{array}{rcl}{H}_{{{{{{{{\rm{inter}}}}}}}}}=&-&{J}_{1}\mathop{\sum}\limits_{ < ij > }{{{{{{{{\bf{M}}}}}}}}}_{{{{{{{{\bf{i}}}}}}}}}^{{{{{{{{\bf{1}}}}}}}}}\cdot {{{{{{{{\bf{M}}}}}}}}}_{{{{{{{{\bf{j}}}}}}}}}^{{{{{{{{\bf{1}}}}}}}}}-{J}_{1}\mathop{\sum}\limits_{ < ij > }{{{{{{{{\bf{M}}}}}}}}}_{{{{{{{{\bf{i}}}}}}}}}^{{{{{{{{\bf{2}}}}}}}}}\cdot {{{{{{{{\bf{M}}}}}}}}}_{{{{{{{{\bf{j}}}}}}}}}^{{{{{{{{\bf{2}}}}}}}}}\\ &-&{J}_{2}\mathop{\sum}\limits_{ < ij > }{{{{{{{{\bf{m}}}}}}}}}_{{{{{{{{\bf{i}}}}}}}}}^{{{{{{{{\bf{1}}}}}}}}}\cdot {{{{{{{{\bf{m}}}}}}}}}_{{{{{{{{\bf{j}}}}}}}}}^{{{{{{{{\bf{1}}}}}}}}}-{J}_{2}\mathop{\sum}\limits_{ < ij > }{{{{{{{{\bf{m}}}}}}}}}_{{{{{{{{\bf{i}}}}}}}}}^{{{{{{{{\bf{2}}}}}}}}}\cdot {{{{{{{{\bf{m}}}}}}}}}_{{{{{{{{\bf{j}}}}}}}}}^{{{{{{{{\bf{2}}}}}}}}}\\ &+&{J}_{4}\mathop{\sum}\limits_{ < ij > }{{{{{{{{\bf{M}}}}}}}}}_{{{{{{{{\bf{i}}}}}}}}}^{{{{{{{{\bf{1}}}}}}}}}\cdot {{{{{{{{\bf{M}}}}}}}}}_{{{{{{{{\bf{j}}}}}}}}}^{{{{{{{{\bf{2}}}}}}}}}-{J}_{5}\mathop{\sum}\limits_{ < ij > }{{{{{{{{\bf{m}}}}}}}}}_{{{{{{{{\bf{i}}}}}}}}}^{{{{{{{{\bf{1}}}}}}}}}\cdot {{{{{{{{\bf{m}}}}}}}}}_{{{{{{{{\bf{j}}}}}}}}}^{{{{{{{{\bf{2}}}}}}}}}\\ &+&{J}_{3}\mathop{\sum}\limits_{ < ij > }{{{{{{{{\bf{M}}}}}}}}}_{{{{{{{{\bf{i}}}}}}}}}^{{{{{{{{\bf{1}}}}}}}}}\cdot {{{{{{{{\bf{m}}}}}}}}}_{{{{{{{{\bf{j}}}}}}}}}^{{{{{{{{\bf{1}}}}}}}}}+{J}_{3}\mathop{\sum}\limits_{ < ij > }{{{{{{{{\bf{M}}}}}}}}}_{{{{{{{{\bf{i}}}}}}}}}^{{{{{{{{\bf{2}}}}}}}}}\cdot {{{{{{{{\bf{m}}}}}}}}}_{{{{{{{{\bf{j}}}}}}}}}^{{{{{{{{\bf{2}}}}}}}}}\end{array}$$

where **m** and **M** are the magnetic moments at each Bi and Mn lattice sites, superscripts 1 and 2 represent the SL layer indices, and the sum is over all the nearest-neighboring lattice sites. In the presence of Mn_Bi_ antisite defects, the energy difference per unit cell between the AFM and FM configurations (Fig. 2c, d) can be expressed as:

2 $${U}_{{{{{{{{\rm{AFM}}}}}}}}}-{U}_{{{{{{{{\rm{FM}}}}}}}}}=2\Bigg({J}_{5}\mathop{\sum}\limits_{ < ij > }{m}_{i}^{1}{m}_{j}^{2}-{J}_{4}\mathop{\sum}\limits_{ < ij > }{M}_{i}^{1}{M}_{j}^{2}\Bigg)$$

This energy difference increases with the amount of Mn-Bi site mixing (the summation of *M* at Mn layer decreases and the summation of *m* at Bi layer increases), eventually leading to a change in the sign of the interlayer magnetic coupling (from AFM to FM). The randomly distributed Mn_Bi_ antisites lead to a spatially inhomogeneous interlayer coupling. As a result, the FM-AFM coexisting ground states are expected to emerge when the energy gain from forming FM domains exceeds the energy cost from forming domain walls. In general, the lattice defect concentration of the MBT family is difficult to precisely control due to the narrow growth temperature window and the large size difference between Mn and Bi ions. Then, in the isostructural MnSb_2_Te_4_ with a larger growth temperature window^44^, we observe the evolution of A-type AFM to FM-AFM coexistence and finally to the FM ground state with varying growth temperature (Supplementary Fig. S12).

### Domain size and distribution characterizations

**Fig. 3:**
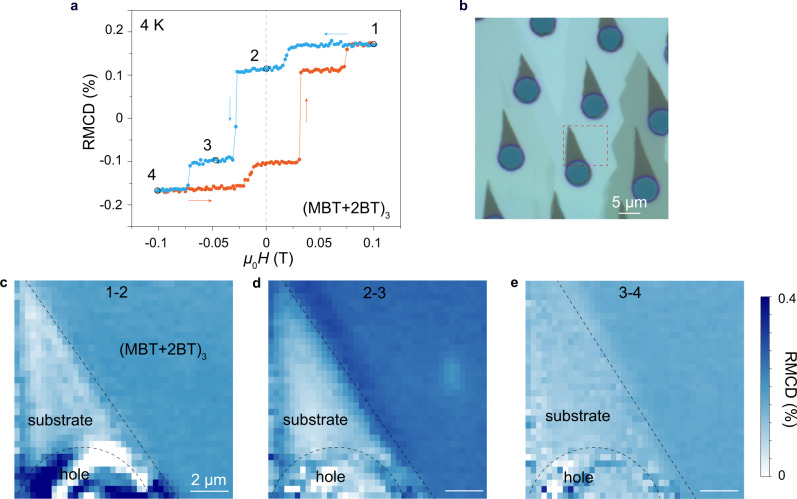
Spatial RMCD mappings of (MBT+2BT)_3_ sample. **a** RMCD signal versus external magnetic field of a 3-SL MnBi_6_Te_10_ flake at 4 K. Four magnetic fields (labeled as 1–4) corresponding to four different spin configurations are selected for RMCD mapping. **b** Optical image of the stepped MnBi_6_Te_10_ sample. The selected area for RMCD mapping is labeled by the dashed red box. **c**–**e** Spatial distributions of the RMCD signal changes in two AFM spin-flip transitions (1–2 and 3–4) and one FM spin-flip transition (2–3). The homogeneous signals indicate the uniform FM-AFM coexistence under the experimental spatial resolution. The bare substrate and etched hole are marked by black dashed lines.

To further evaluate the domain sizes of the FM and AFM components, we characterize the magnetic spatial homogeneity by RMCD mapping. In the typical RMCD-*μ*_0_*H* curve of a 3-SL MnBi_6_Te_10_ sample (Fig. 3a), we map the RMCD signals in a selected area (Fig. 3b) under four selected magnetic fields (0.1, 0, −0.05, and −0.1 T, respectively) corresponding to four different spin configurations (see Supplementary Fig. S13 for MnBi_4_Te_7_ sample). Changes in the RMCD signal at the two AFM spin-flip transitions (Fig. 3c, e) and the FM spin-flip transition (Fig. 3d) are uniform across the scanned sample area, indicating homogeneous FM-AFM coexistence at a spatial resolution limited by the laser spot size of ~2 μm. The small domain size of the FM and AFM components is consistent with the large number of Mn-Bi site mixing characterized by SC-XRD (see Tables I and II in Supplementary Note VIII). It should be noted that these spin-flip transitions, especially for the FM spin-flip transition at *H*_c_, are quite sharp despite the FM-AFM spatial inhomogeneity. In an inhomogeneous system, the sharp transition field suggests that its magnetic reversal is dominated by the nucleation of reverse domain and the subsequent domain wall motion processes (see detailed discussions in Supplementary Note VII and Supplementary Fig. S14).

### Tunable exchange bias effect

**Fig. 4:**
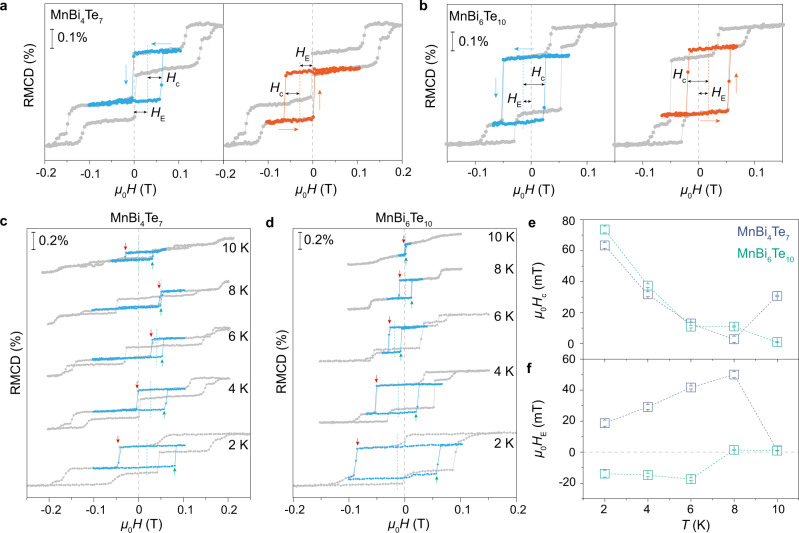
Exchange bias effects of the FM component in MnBi_4_Te_7_ and MnBi_6_Te_10_ samples. **a**, **b** Exchange bias effects of the FM component in the 6-SL MnBi_4_Te_7_ (**a**) and 6-SL MnBi_6_Te_10_ (**b**) measured at 4 K. The direction of the exchange bias can be tuned by the historical polarization field (blue for positive polarization field and orange for negative polarization field). The exchange bias effect observed in MnBi_6_Te_10_ is opposite to that of MnBi_4_Te_7_. Colored dashed lines represent *H*_E_. **c**, **d** Temperature-dependent exchange bias effects of MnBi_4_Te_7_ and MnBi_6_Te_10_ after initialized by positive polarization field, with the full hysteresis loops being presented as the references (gray lines). The red and green arrows represent the *H*_c–_ and *H*_c+_ for the biased hysteresis loop, respectively, and the blue dashed lines represent *H*_E_. **e**, **f** Evolution of *H*_c_ and *H*_E_ with temperature in MnBi_4_Te_7_ (blue squares) and MnBi_6_Te_10_ (green squares).

The FM-AFM coexisting ground state provides us with a unique system to study exchange bias effects. The large-field full hysteresis loops at 4 K (gray data in Fig. 4a–d) are plotted as references for the minor hysteresis loops of the FM components. Historically polarized by a positive saturation magnetic field, the minor hysteresis loop of the FM component shifts to the right side in MnBi_4_Te_7_ sample, namely a positive exchange bias (blue data in Fig. 4a). By contrast, polarized by a negative saturation magnetic field, then the minor hysteresis loop of the FM component shifts to the left, indicating a negative exchange bias (orange data in Fig. 4a). The direction of this exchange bias can be easily tuned by the historical polarization field and does not require warming and field cooling processes. Moreover, this exchange bias is rather stable under multiple back-and-forth magnetic field sweeps, with no training effect (Supplementary Fig. S15). The exchange bias effect observed in MnBi_6_Te_10_ is opposite to that of MnBi_4_Te_7_ (Fig. 4b), possibly due to the different magnetic interactions at the FM/AFM interfaces because of the different interlayer coupling strengths (see discussion following Supplementary Fig. S10).

As the temperature increases, the coercive field *H*_c_ for the biased hysteresis loop, defined as 1/2(*H*_c+_ − *H*_c−_), gradually shrinks and the exchange bias field *H*_E_, defined as 1/2(*H*_c+_ + *H*_c−_), slightly increases (Fig. 4c–f). The *H*_c_ shows an abnormal increase at 10 K for MnBi_4_Te_7_ and 8 K for MnBi_6_Te_10_, respectively, accompanied by the vanishment of the exchange bias effect. In typical AFM and FM heterostructure systems, the exchange bias effect occurs only when (*K*_AFM_*t*_AFM_)/*J*_int_ ≥ 1, where *K*_AFM_ is the anisotropy energy of the AFM component, *t*_AFM_ is the thickness of the AFM component, and *J*_int_ is the exchange coupling between the AFM and FM components. As the temperature increase, the *K*_AFM_ in the MBT system decreases, and eventually, the AFM pinning layer flips collectively with the FM spins, resulting in the vanishment of the exchange bias and the abnormal increase in the coercive field. At high temperatures, the two AFM spin-flip transitions lead to identical RMCD signal changes, also confirming the collective flipping of the AFM and FM components during the FM spin-flip transition. The EB effect can be used reliably when the temperature, number of layers, and especially defect concentration are precisely controlled.

## Discussion

In summary, we systematically study the magnetism of atomically thin MnBi_4_Te_7_ and MnBi_6_Te_10_ flakes in the parameter space of layer number, temperature and applied magnetic field using RMCD spectroscopy. The complex FM-AFM coexisting ground state is observed and verified. The weakened interlayer coupling and inhomogeneously distributed ubiquitous Mn-Bi site mixing have been attributed to result in such a unique magnetic ground state. A tunable exchange bias effect without the assistance of field cooling process is observed in MnBi_4_Te_7_ and MnBi_6_Te_10_, arising from the coupling between the FM and AFM components. Instead of fabricating heterostructures with magnetically ordered systems, such as AFM and FM^45–48^, we obtained the magnetic ground state of FM-AFM coexistence in a single material by a simple exfoliation process, while the crystal structure remained intact. This FM-AFM coexisting ground state expands EB phenomenon and provides design principles and materials for spintronic devices. The EB effect observed in the MnBi_4_Te_7_ and MnBi_6_Te_10_ system may not have a high enough critical temperature for spintronics in the near future, but the mechanism we learn from them actually helps us understand the principle and therefore design material systems with higher critical temperatures. Using sophisticated techniques, synthetic antiferromagnets composed of two or more ferromagnetic layers separated by nonmagnetic spacers can be precisely prepared^49^. Due to the weak interlayer exchange coupling in synthetic antiferromagnets, introducing spatial inhomogeneity in thickness of the spacer layer or disorders can therefore tune the interaction and form an FM-AFM coexisting ground state, allowing for precise manipulation of the exchange bias effect in this system. By unraveling the puzzling magnetic states in MnBi_4_Te_7_ and MnBi_6_Te_10_ flakes, our findings pave the way for further investigation of quantum phenomena intertwined with the magnetic orders.

## Methods

### Crystal growth and magnetic characterizations

MnBi_2_Te_4_(Bi_2_Te_3_)_*n*_ (*n* = 1, 2) single crystals were grown by flux method^34^. Mn powder, Bi lump and Te lump were weighed with the ratio of Mn:Bi:Te = 1:8:13 (MnTe:Bi_2_Te_3_ = 1:4). The mixture was loaded into a corundum crucible sealed into a quartz tube. The tube was then placed into a furnace and heated to 1100 °C for 20 h to allow sufficient homogenization. After a rapid cooling to 600 °C at 5 °C/h, the mixture was cooled slowly to 585 °C (581 °C) at 0.5 °C/h for MnBi_4_Te_7_ (MnBi_6_Te_10_) and kept at this temperature for 2 days. Finally, the single crystals were obtained after centrifuging. The centimeter-scale plate-like MnBi_4_Te_7_ and MnBi_6_Te_10_ single crystals can be easily exfoliated. Magnetic measurements of MnBi_4_Te_7_ and MnBi_6_Te_10_ single crystals were measured by the vibrating sample magnetometer (VSM) option in a Quantum Design Physical Properties Measurement System (PPMS-9 T). The temperature-dependent magnetization measurements are described in detail in Supplementary Note I.

### Preparation of the ultra-thin samples

The MnBi_4_Te_7_ and MnBi_6_Te_10_ flakes with different thicknesses were first mechanically exfoliated on a polydimethylsiloxane (PDMS) substrate by the Scotch tape method. The exfoliated samples on PDMS substrates were then dry-transferred onto 285 nm SiO_2_/Si substrates with evaporated gold films. Then, a layer of PMMA was spin-coated on the thin flakes for protection.

### AFM characterization

The thickness of the ultra-thin samples was verified by the atomic force microscopy characterization using the Oxford Cypher S system in tapping mode. According to the height line profiles, the MnBi_4_Te_7_ and MnBi_6_Te_10_ were confirmed to possess an alternated lattice structure of BT (1 nm) + MBT (1.4 nm) and BT (1 nm) + BT (1 nm) + MBT (1.4 nm). See more details in Supplementary Note II.

### RMCD measurements

The RMCD measurements were performed based on the Attocube closed-cycle cryostat (attoDRY2100) down to 1.6 K and up to 9 T in the out-of-plane direction. The linearly polarized light of 633 nm HeNe laser was modulated between left and right circular polarization by a photoelastic modulator (PEM) and focused on the sample through a high numerical aperture (0.82) objective. The reflected light was detected by a photomultiplier tube (THORLABS PMT1001/M). The magnetic reversal under external magnetic field was detected by the RMCD signal determined by the ratio of the a.c. component of PEM at 50.052 kHz and the a.c. component of chopper at 779 Hz (dealt by a two-channel lock-in amplifier Zurich HF2LI). The errors of ratio of FM and AFM components are determined by the instability of the data acquired during RMCD measurements.

### STEM characterization

Samples for cross-sectional investigations were prepared by standard lift-out procedures using an FEI Helios NanoLab G3 CX-focused ion beam system. To minimize sidewall damage and make the samples sufficiently thin to be electronically transparent, final milling was carried out at a voltage of 5 kV and a fine milling at 2 kV. Aberration-corrected STEM imaging was performed using a Nion HERMES-100 operating at an acceleration voltage of 60 kV and a probe forming semi-angle of 32 mrad. HAADF images were acquired using an annular detector with a collection semi-angle of 75–210 mrad. EELS measurements were performed using a collection semi-angle of 75 mrad, an energy dispersion of 0.3 eV per channel, and a probe current of ~20 pA. The Mn-*L* (640 eV) and Te-*M* (572 eV) absorption edges were integrated for elemental mapping after background subtraction. The original spectrum images were processed to reduce random noise using a principal component analysis (PCA) tool. HAADF image simulations were computed using the STEM_CELL software simulation package matching the microscope experimental settings described above and using a supercell with a thickness ~20 nm.

## Supplementary information

Supplementary Information

## Data Availability

The source data generated in this study have been deposited in the Zenodo database under the accession code https://zenodo.org/badge/latestdoi/541886998. Source Data are provided with this paper.

## Source data

Source Data
