# Supplementary material for: Ferromagnetic-antiferromagnetic coexisting ground state and exchange bias effects in MnBi4Te7 and MnBi6Te10
Source: Nat Commun. 2022 Dec 10;13:7646. doi: 10.1038/s41467-022-35184-7 (PMC9741634; doi:10.1038/s41467-022-35184-7)
Supplement: Supplementary file 1 — Supplementary Information [file 41467_2022_35184_MOESM1_ESM.pdf]

Supplementary information for Ferromagnetic–antiferromagnetic coexisting ground states and exchange bias effects in $MnBi_4Te_7$ and $MnBi_6Te_{10}$

Xiaolong Xu,[1,2,3] Shiqi Yang,[1,4] Huan Wang,[5] Roger Guzman,[6] Yaozheng Zhu,[1] Yuxuan Peng,[1] Zhihao Zang,[1] Ming Xi,[5] Shagnjie Tian,[5] Yanping Li,[1] Hechang Lei,[5,7] Zhaochu Luo,[1] Jinbo Yang,[1] Yeliang Wang,[2] Tianlong Xia,[5] Wu Zhou,[6,8] Yuan Huang,[2,9] and Yu Ye[1,3,10]

[1]*State Key Laboratory for Mesoscopic Physics and Frontiers Science Center for Nano-optoelectronics, School of Physics, Peking University, Beijing 100871, China*
[2]*School of Integrated Circuits and Electronics, MIIT Key Laboratory for Low-Dimensional Quantum Structure and Devices, Beijing Institute of Technology, Beijing, 100081, China*
[3]*Collaborative Innovation Center of Quantum Matter, Beijing 100871, China*
[4]*Academy for Advanced Interdisciplinary Studies, Peking University, Beijing 100871, China*
[5]*Department of Physics, Renmin University of China, Beijing 100872, China*
[6]*School of Physical Sciences, University of Chinese Academy of Sciences, Beijing 100049, China*
[7]*Laboratory for Neutron Scattering, Department of Physics, Renmin University of China, Beijing 100872, China*
[8]*CAS Centre for Excellence in Topological Quantum Computation, University of Chinese Academy of Sciences, Beijing 100049, China*
[9]*Advanced Research Institute of Multidisciplinary Science, Beijing Institute of Technology, Beijing 100081, China*
[10]*Yangtze Delta Institute of Optoelectronics, Peking University, Nantong 226010 Jiangsu, China*

Contents:

Supplementary Note I.
Characterizations of bulk $MnBi_4Te_7$ and $MnBi_6Te_{10}$ crystals.
Supplementary Note II.
Atomic force microscopy measurements of few-layer $MnBi_4Te_7$ and $MnBi_6Te_{10}$ flakes.
Supplementary Note III.
RMCD measurements of few-layer $MnBi_4Te_7$ and $MnBi_6Te_{10}$ flakes with different thicknesses.
Supplementary Note IV.
Macrospin model of a specific FM-AFM coexisting states of five-layer $MnBi_4Te_7$ and $MnBi_6Te_{10}$.
Supplementary Note V.
Cross-sectional STEM-EELS characterizations of $MnBi_4Te_7$.
Supplementary Note VI.
RMCD measurements of few-layer $MnSb_2Te_4$ samples from three synthesized crystals.
Supplementary Note VII.
Discussions of the FM-AFM domain distribution
Supplementary Note VIII.
Single-crystal X-ray diffraction characterizations of $MnBi_4Te_7$ and $MnBi_6Te_{10}$.
Supplementary Note IX.
The exchange bias effect of the FM component.

Supplementary Note I. Characterizations of bulk $MnBi_4Te_7$ and $MnBi_6Te_{10}$ crystals

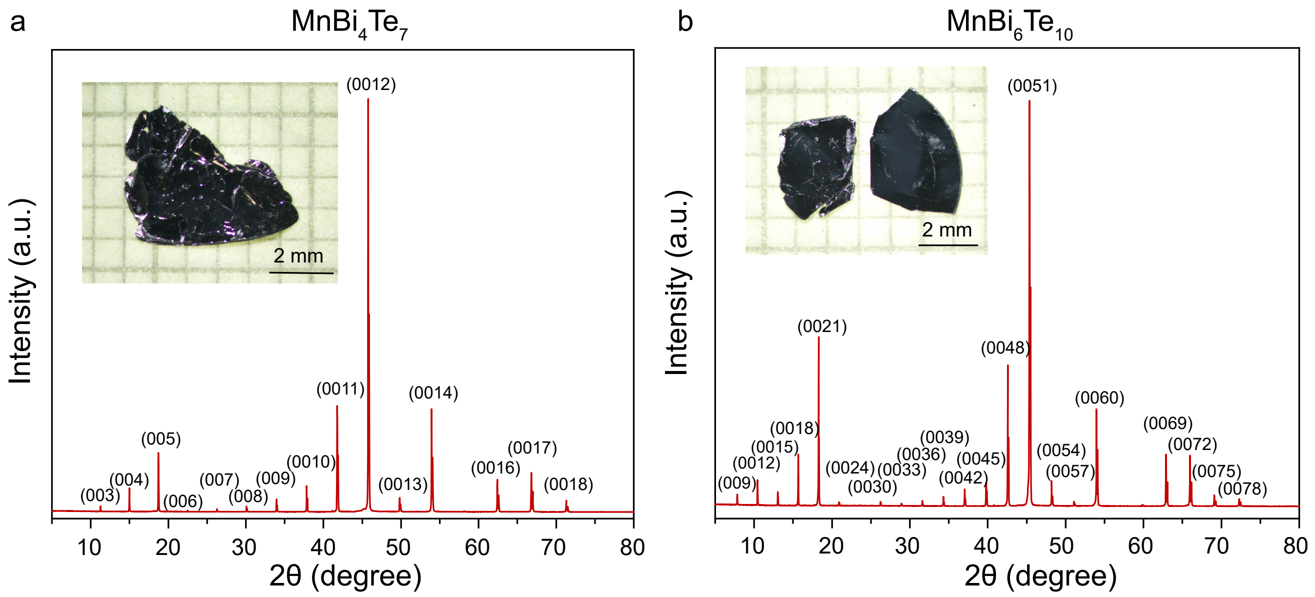

FIG. S1. **X-ray diffraction patterns of bulk $MnBi_4Te_7$ and $MnBi_6Te_{10}$ crystals.** The observed sharp (00l) peaks imply the high quality of the synthesized $MnBi_4Te_7$ and $MnBi_6Te_{10}$ crystals. Insets: optical images of the grown $MnBi_4Te_7$ and $MnBi_6Te_{10}$ bulk crystals.

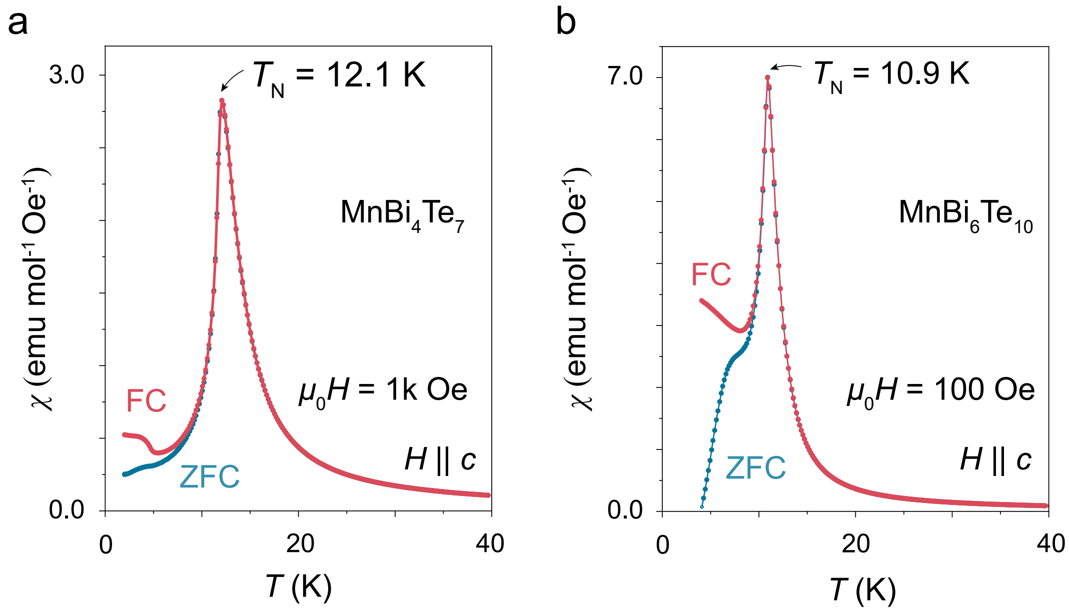

FIG. S2. **Magnetic measurements of bulk $MnBi_4Te_7$ and $MnBi_6Te_{10}$ bulk crystals.** (a) The zero-field-cooled (ZFC) and field-cooled (FC) magnetic susceptibilities of $H \parallel c$ (χ^c) of $MnBi_4Te_7$ crystals measured at 1 kOe, which indicate an PM to AFM transition at a Néel temperature of 12.1 K. An obvious bifurcation between the FC and ZFC curves was observed at temperatures below T_N, which also help confirm the FM component in the AFM states. (b) The ZFC and FC magnetic susceptibilities of $H \parallel c$ (χ^c) of $MnBi_6Te_{10}$ crystals measured at 100 Oe, showing a Néel temperature of 10.9 K, and a more distinguished bifurcation between FC and ZFC curves was observed below a temperature of around 8 K (which were also obseved in [1, 2]). The bifurcations of the ZFC and FC curves at temperatures slightly below the Néel temperature indicate the FM-AFM coexisting magnetic order.

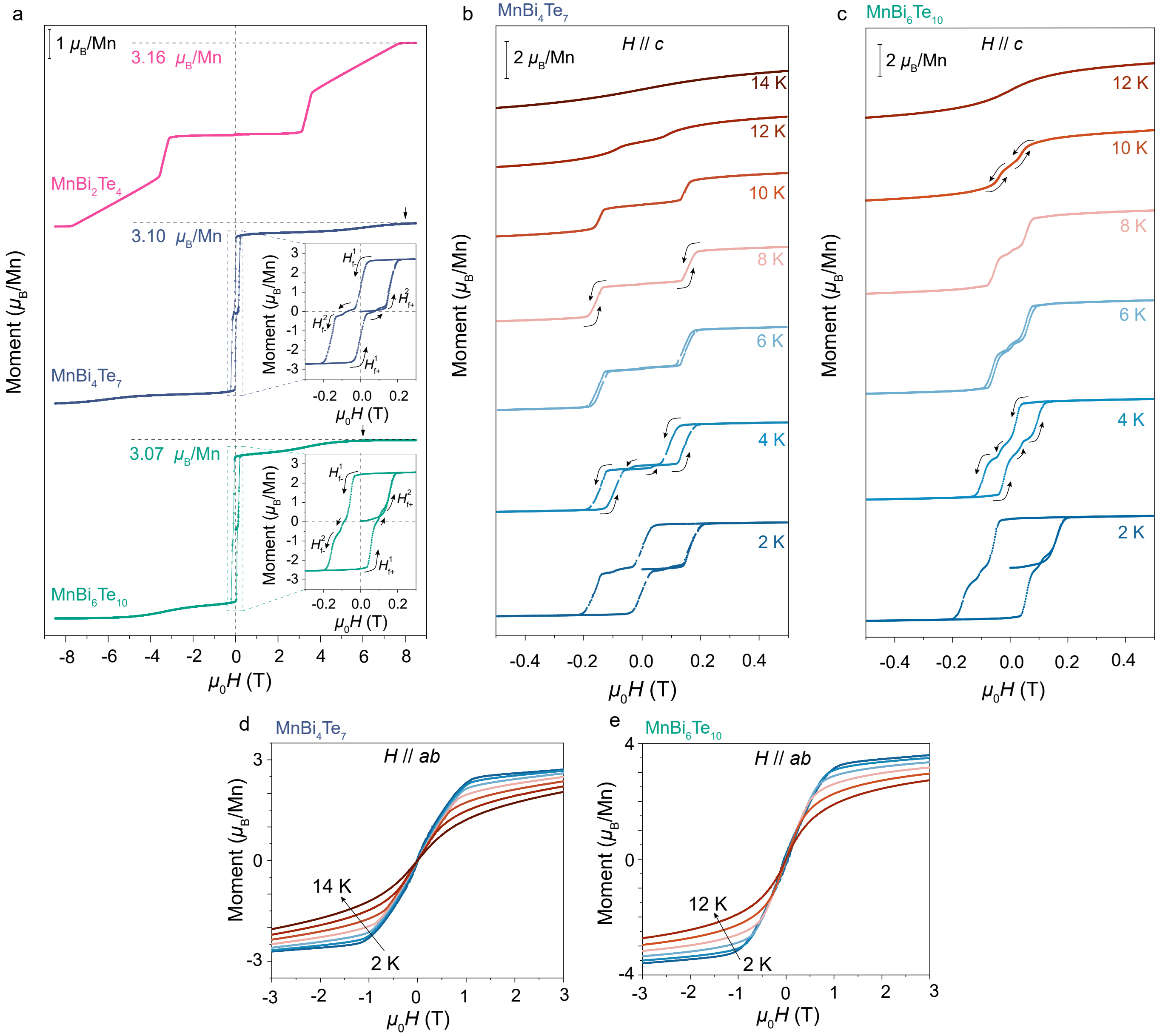

FIG. S3. **Magnetic measurements of bulk MnBi₂Te₄, MnBi₄Te₇, and MnBi₆Te₁₀ crystals.** (a) $M-H$ curves for $H \parallel c$ of $MnBi_2Te_4$, $MnBi_4Te_7$ and $MnBi_6Te_{10}$ taken at 2 K up to 8.5 T. Insets: the zoomed-in images at small magnetic fields. The horizontal dashed lines marks the measured saturation magnetic moment of the $MnBi_2Te_4$, $MnBi_4Te_7$, and $MnBi_6Te_{10}$ crystals. The black arrows represent that the magnetic moment in the QL flip to align with the magnetic filed at a magnetic filed of about 8 T and 6 T in $MnBi_4Te_7$ and $MnBi_6Te_{10}$, respectively. The black arrows in the insets mark the multi-step magnetic transitions. (b, c) Temperature-dependent $M-H$ curves for $H \parallel c$ of $MnBi_4Te_7$ and $MnBi_6Te_{10}$. The black arrows mark the multi-step magnetic transitions. (d, e) Temperature-dependent $M-H$ curves for $H \parallel ab$ of $MnBi_4Te_7$ and $MnBi_6Te_{10}$.

Scrutiny of the $M-H$ curve at 2 K under $H \parallel c$ (Fig. S3a), we find that $MnBi_4Te_7$ ($MnBi_6Te_{10}$) undergoes a spin-flip transition at a very low magnetic field and quickly enters the forced ferromagnetic (FM) state at about 0.25 T (0.21 T). That is in sharp contrast with $MnBi_2Te_4$, where the spin-flop transition occurs at about 3.5 T and its magnetic moment finally saturates under an external magnetic field larger than 8 T. We estimate the interlayer antiferromagnetic coupling J_c and single-ion anisotropy D based on the Stoner-Wohlfarth model[3]. Unlike $MnBi_2Te_4$, which has comparable anisotropy and interlayer exchange energy, spin-flop transitions are absent in $MnBi_4Te_7$ ($MnBi_6Te_{10}$), instead, spin-flip transitions are found at H_f^c of about 0.22 T (0.19 T) at $H \parallel c$ at 2 K (Figs. S3b and c).The spin-flip

field can be given by $g\mu_B H_c^{flip} = g\mu_B H_f^c = zSJ_c$, thus giving

$$SJ_c = g\mu_B H_f^c / z, \tag{1}$$

$$SD = g\mu_B (H_{sat}^{ab} - H_f^c)/2 \tag{2}$$

where g =2 is the Landé g factor, $S = 5/2$, and $z = 2$ ($z = 6$) is the Mn nearest neighbors in adjacent septuple layers of $MnBi_4Te_7$ ($MnBi_6Te_{10}$). Here, H_{sat}^{ab} refers to the saturation field when H is applied parallel to the ab plane. For $MnBi_4Te_7$, $H_c^{flip} \approx 0.22$ T and $H_{sat}^{ab} \approx 1.2$ T, from which we obtain $SJ_c \approx 0.0127$ meV and $SD \approx 0.0440$ meV. For $MnBi_6Te_{10}$, $H_c^{flip} \approx 0.19$ T and $H_{sat}^{ab} \approx 1.1$ T, from which we obtain $SJ_c \approx 0.0037$ meV and $SD \approx 0.0417$ meV. The anisotropy energies of $MnBi_4Te_7$ and $MnBi_6Te_{10}$ are of the same order of magnitude as that of $MnBi_2Te_4$, but the interlayer coupling values of $MnBi_4Te_7$ and $MnBi_6Te_{10}$ are almost 1 to 2 orders of magnitudes smaller than that of $MnBi_2Te_4$[4–6], indicating a greatly reduced interlayer coupling from $MnBi_2Te_4$ to $MnBi_4Te_7$ and $MnBi_6Te_{10}$. Thus, the magnetic moment will not tend to be perpendicular to the direction of the applied field to cause the spin-flop transition, but will flip to be parallel to the direction of the field under a small critical field. It is worth noting that at low temperatures, the $MnBi_4Te_7$ and $MnBi_6Te_{10}$ crystals show non-zero magnetization under zero field, and the magnetic reversal is completed through three sluggish spin-flip transitions (marked by the arrows in insets of Fig. S3a). The nonzero moments of the plateaus imply that there may be some residual FM states in the AFM state. These inconspicuous steps disappear above 6 K in $MnBi_4Te_7$ (above 8 K in $MnBi_6Te_{10}$) (Fig. S3b and Fig. S3c).

Supplementary Note II. Atomic force microscopy measurements of few-layer $MnBi_4Te_7$ and $MnBi_6Te_{10}$ flakes

Before atomic force microscopy measurements, the PMMA covering on the $MnBi_4Te_7$ and $MnBi_6Te_{10}$ samples were removed using acetone, and then the samples were thoroughly rinsed with isopropanol (IPA). The residual particles on the surface were cleaned by the contact mode of the instrument to obtain accurate step thicknesses. The detailed results are shown in the following.

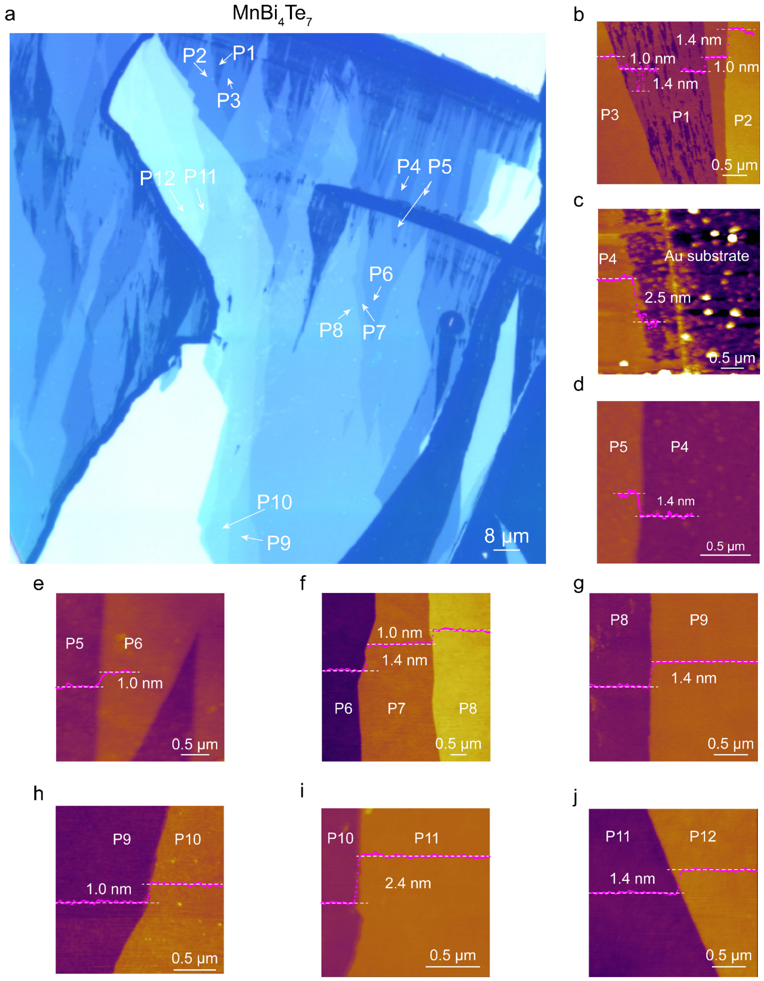

FIG. S4. **Optical image and corresponding atomic force microscopy height images of few-layer $MnBi_4Te_7$ flakes.** The height line profiles are superimposed in each atomic force microscopy height images, showing that the thickesses of steps are ∼1.0 nm (Bi_2Te_3, BT), ∼1.4 nm ($MnBi_2Te_4$, MBT) or ∼2.4 nm (MBT+BT or BT+MBT, which can be confirmed by the adjacent layer).

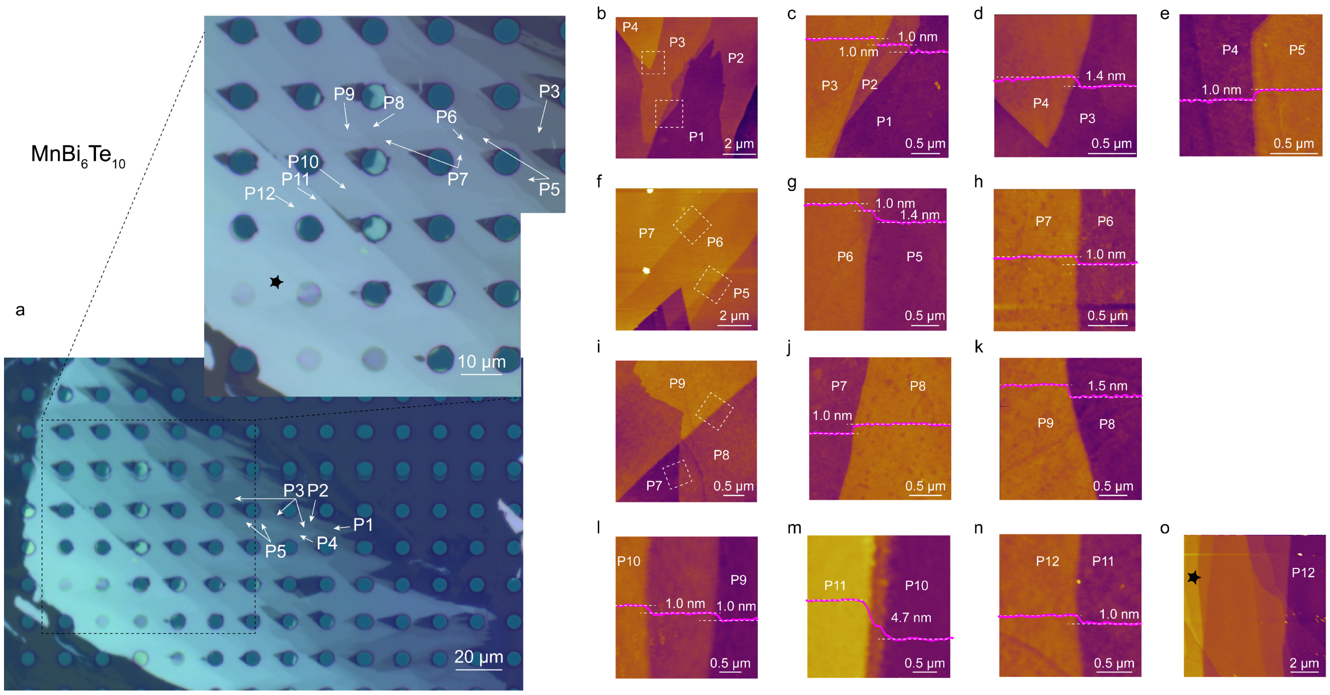

FIG. S5. **Optical image and corresponding atomic force microscopy height images of few-layer $MnBi_6Te_{10}$ flakes.** The height line profiles are superimposed in each atomic force microscopy height images, showing that the thicknesses steps are ∼1.0 nm (BT), ∼1.4 nm (MBT) or ∼4.7 nm (MBT+BT+BT+MBT). The distribution of step thickness is consistent with the superlattice structure of $MnBi_6Te_{10}$.

Supplementary Note III. RMCD measurements of few-layer $MnBi_4Te_7$ and $MnBi_6Te_{10}$ flakes with different thicknesses

The RMCD measurements of all the few-layer $MnBi_4Te_7$ and $MnBi_6Te_{10}$ samples at 2 K are presented in this section. There are two kinds of terminations in $MnBi_4Te_7$, namely MBT and MBT+BT at the outermost layer, which are drawn in green and orange colors, respectively (Fig. S6). For $MnBi_6Te_{10}$, there are three kinds of terminations, namely MBT, MBT+BT and MBT+BT+BT at the outermost layer. The BT+BT protected samples were presented in the main text, thus here we only show MBT and MBT+BT terminations drawn in green and orange colors (Fig. S7).

The temperature-dependent RMCD measurements of $MnBi_6Te_{10}$ and $MnBi_4Te_7$ samples are also shown in this part. With the increasing temperature, the hysteresis loop of 1 SL $MnBi_6Te_{10}$ shrinks and disappears at 10 K, indicating an FM to PM phase transition at 8-10 K (Fig. S8a). Compared with the intrinsic 1-SL $MnBi_2Te_4$ (T_C = 14.5 K), the decreased T_C may be due to the increased Bi_{Mn} antisite defects, as the the intralayer exchange coupling decreases with the increase of the average distance between the occupied intrinsic Mn atoms. The 6 SL-$MnBi_6Te_{10}$ posseses similar behaviors as that of 3 SLs presented in the main text (Fig. S8b). For temperature-dependent measurements of $MnBi_4Te_7$ samples (Fig. S9), we drew the descending and ascending curves in blue and orange, respectively. The temperature-dependent measurements all show increasingly pronounced multi-step spin-flip transitions, which are similar to $MnBi_6Te_{10}$. However, there are still differences between the $MnBi_6Te_{10}$ and $MnBi_4Te_7$, which are discussed in the exchange bias sections in the main text.

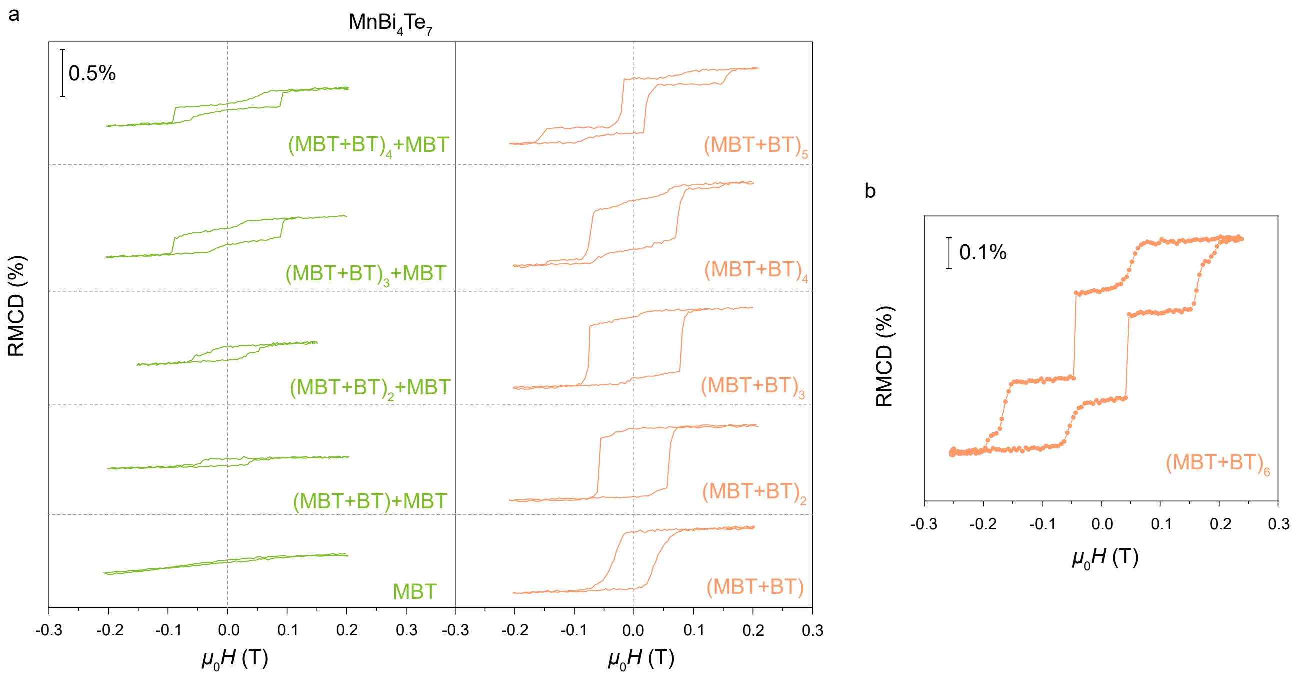

FIG. S6. **RMCD measurements of $MnBi_4Te_7$ flakes with different thicknesses.** Two different terminations, namely MBT and MBT+BT, are characterized. The FM-AFM coexisting ground state appears and become more and more obvious with the number of MBT layer increase, regardless of termination.

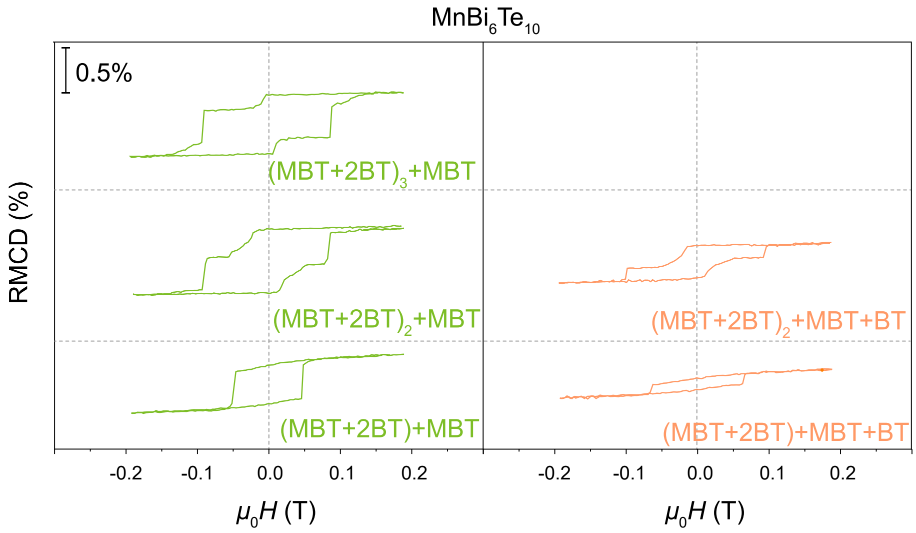

FIG. S7. **RMCD measurements of $MnBi_6Te_{10}$ flakes with different thickness.** Three different terminations, namely MBT, MBT+BT and MBT+BT+BT (also see Fig. 2c in the main text), are characterized. The FM-AFM coexisting ground state appears and become more and more obvious with number of MBT layer increase, regardless of termination.

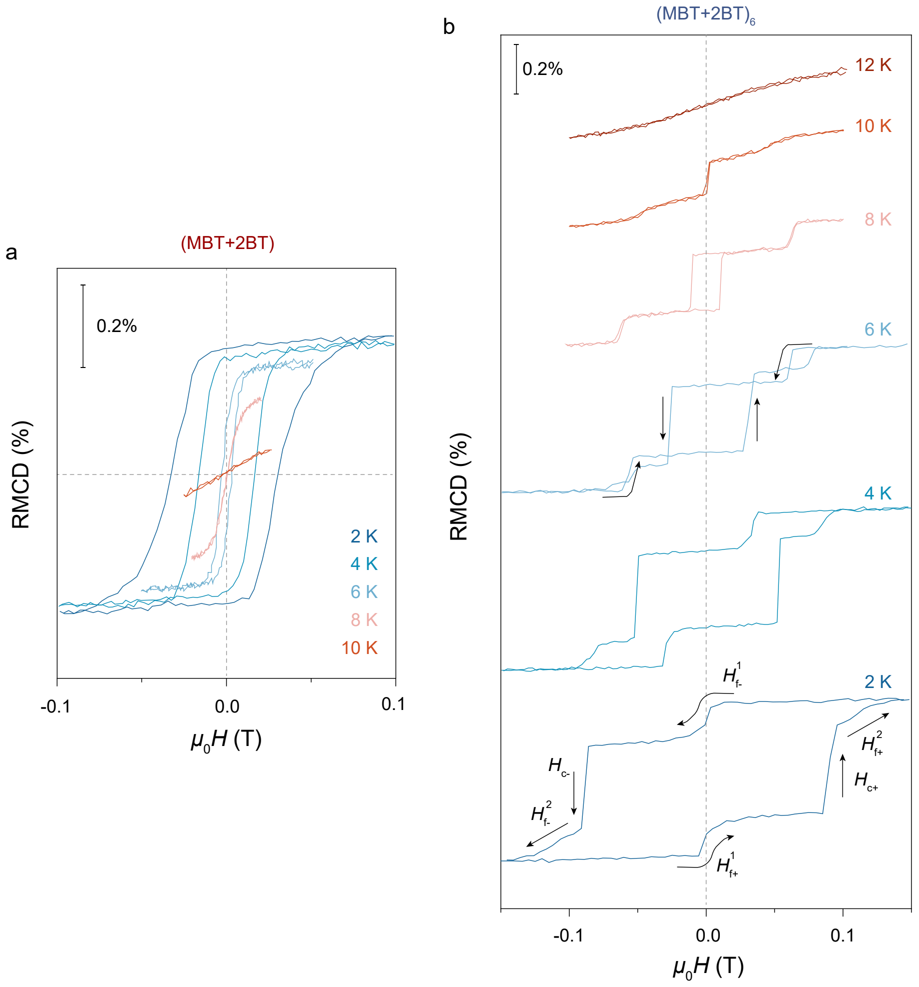

FIG. S8. **Temperature-dependent RMCD measurements of $MnBi_6Te_{10}$ flakes.** Temperature-dependent RMCD measurements of $(MBT + 2BT)$ (a) and $(MBT + 2BT)_6$ (b) samples. With increasing temperature, the hysteresis loop of $(MBT + 2BT)$ shrinks and disappears at 10 K, indicating an FM to PM phase transition. (b) RMCD sweeps for the $(MBT + 2BT)_6$ flake at a temperature range that passes through its T_N. The behaviors are very similar to those of $(MBT + 2BT)_3$ flakes shown in Fig. 1d in the main text, with slightly different spin-flip fields.

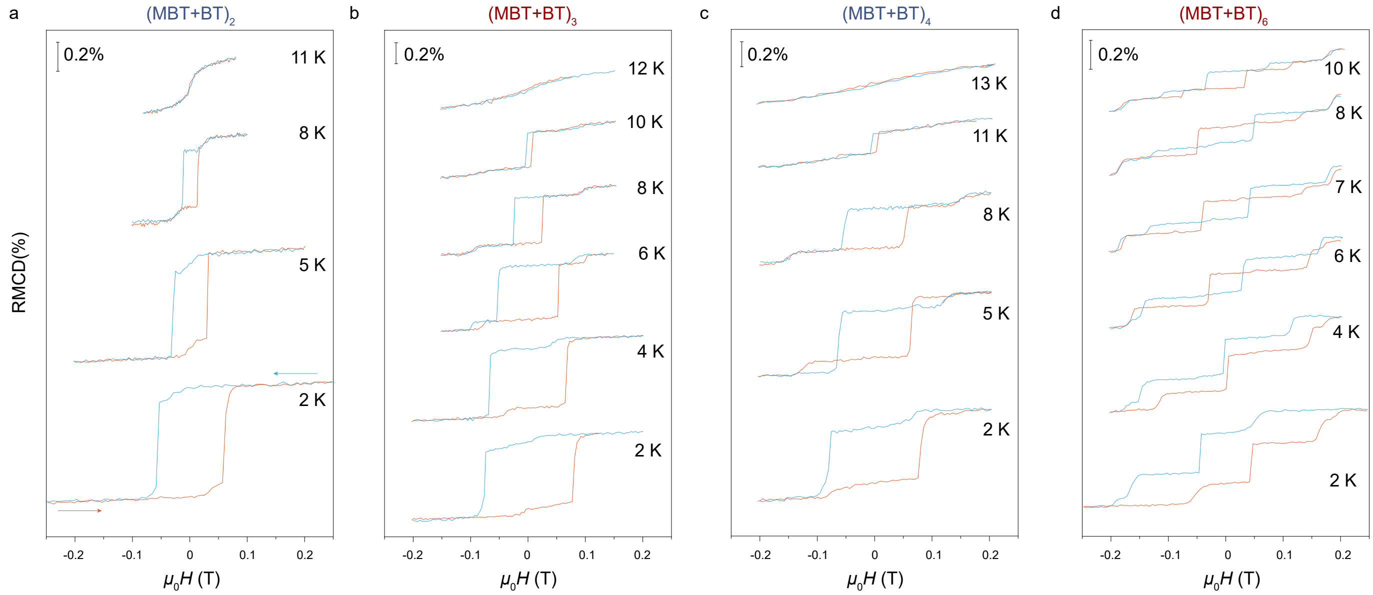

FIG. S9. **Temperature-dependent RMCD measurements of $MnBi_4Te_7$ flakes.** The magnetic ascending and descending sweeping curves were presented in orange and blue. For $(MBT+BT)_6$, H_{c-} first increases from negative to positive and then jumps back to negative with increasing temperature, signifying a coupling between the FM and AFM components.

Supplementary Note IV. Macrospin model of a specific AFM-FM coexisting states of five-layer $MnBi_4Te_7$ and $MnBi_6Te_{10}$

The mixture of the interlayer AFM and FM coupling endows rich hysteresis behaviors in van der Waals magnets. Different ratios between AFM and FM couplings would result in distinct hysteresis loops. Here, we build a five-layer macrospin model to interpret the hysteresis behavior in two materials of $MnBi_4Te_7$ and $MnBi_6Te_{10}$ which have different AFM/FM ratios and exhibit distinct hysteresis loops.

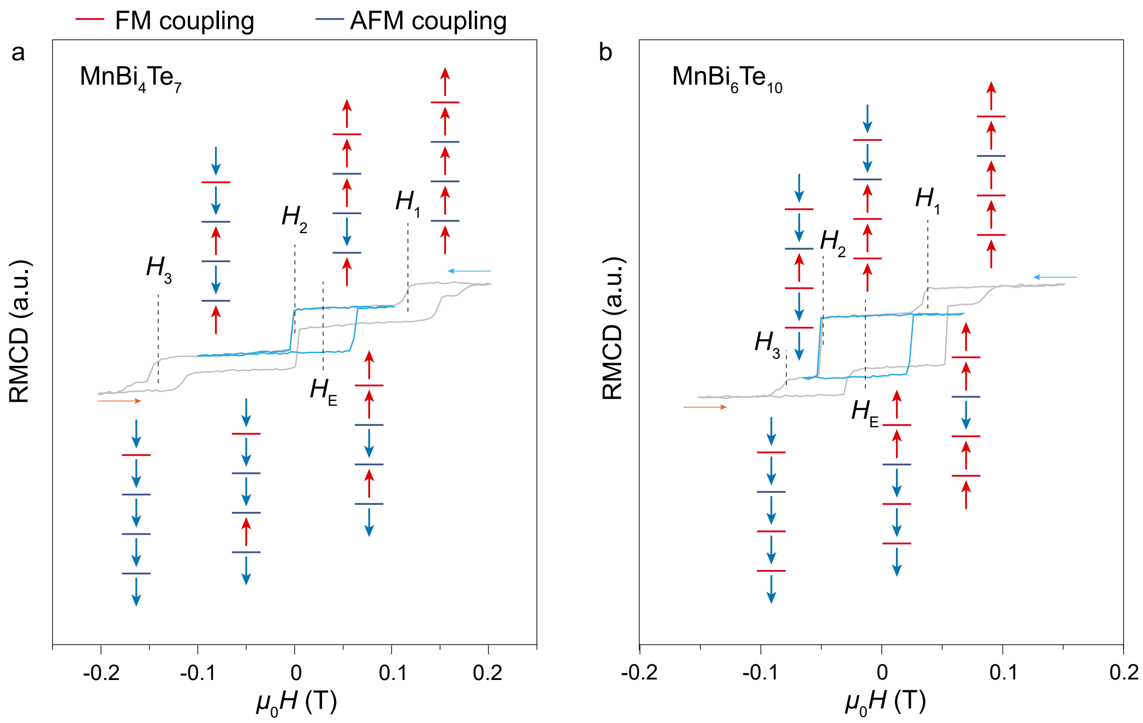

FIG. S10. **Macrospin model of a specific AFM-FM coexisting states of five-layer $MnBi_4Te_7$ and $MnBi_6Te_{10}$.** The inset arrows denote the spin orientation of each SL by the red (↑) and blue (↓) colors. The inter-SL coupling are labled by the horizontal red (FM) and blue (AFM) lines between the SLs.

For the case of $MnBi_4Te_7$ that is supposed to have a high AFM/FM ratio and strong interlayer AFM coupling strength (Fig. S10a), the top two magnetic moments are set to be FM coupled while the rest are set to be AFM coupled. When the magnetic field decreases from the positive saturation magnetic field, some of the magnetic moments flips as the interlayer AFM coupling prefers magnetic moment in the neighboring layer to be antiparallel, forming a step in the hysteresis loop. Because the AFM coupling strength is relatively strong, the switching magnetic field is high and Zeeman energy would keep most of the magnetic moment parallel to the magnetic field. Hence, only the magnetic moment in the middle of AFM region would be flipped. When the magnetic field is reversed, the magnetic moment in the FM region is flipped. Due to the magnetic configuration on the FM/AFM boundary, the minor hysteresis loop of FM region is shifted to the right side, indicating a positive exchange bias (Fig. 4a in main text). Further increase of magnetic field would saturate all magnetic moment in the negative direction. The hysteresis behavior under the magnetic field sweeping in the positive direction is reciprocal to that under the magnetic field sweeping in the negative direction. The minor hysteresis loop of FM region is then shifted to the left side, indicating a negative exchange bias (Fig. 4a in main text).

All the values in the hysteresis loop can be given by:

$$\begin{aligned} H_1 &= \frac{4J_{\text{AFM}} - \Delta E_1}{2m} \\ H_2 &= \frac{2J_{\text{AFM}} - \Delta E_2}{4m} \\ H_{\text{E}} &= \frac{J_{\text{AFM}}}{m} \\ H_3 &= \frac{-6J_{\text{AFM}} - \Delta E_3}{4m} \end{aligned} \tag{3}$$

where, m is magnetic moment of the macrospin, ΔE_1, ΔE_2, and ΔE_3 are the spin-flip barriers for each transition respectively. The strength of J_{AFM} can be estimated by H_{E}.

For the case of $MnBi_6Te_{10}$ that is supposed to have a low AFM/FM ratio and weak interlayer AFM coupling strength (Fig. S10b), one pair of magnetic moments is set to be AFM coupled while the rest are set to be FM coupled. Similar to the case of $MnBi_4Te_7$, when the magnetic field decreases from the positive saturation magnetic field, some of the magnetic moments flips as the interlayer AFM coupling prefers magnetic moment in the neighboring layer to be antiparallel, forming a step in the hysteresis loop. Because the AFM coupling strength is relatively weak, the switching magnetic field is low and the adjacent FM coupled magnetic moment is flipped as well. When the magnetic field is reversed, the magnetic moment in the FM region is flipped. As the third layer of magnetic moment is pointed to up and is FM coupled to the magnetic moments in the FM region, the minor hysteresis loop of FM region is shifted to the left side, indicating a negative exchange bias. Further increase of magnetic field would saturate all magnetic moment in the negative direction. The hysteresis behavior under the magnetic field sweeping in the positive direction is reciprocal to that under the magnetic field sweeping in the negative direction. The minor hysteresis loop of FM region is then shifted to the right side, indicating a positive exchange bias (Fig. 4b in the main text).

All the values in the hysteresis loop can be given by:

$$\begin{aligned} H_1 &= \frac{2J_{\text{AFM}} - \Delta E_1}{4m} \\ H_2 &= \frac{-2J_{\text{FM}} - \Delta E_2}{4m} \\ H_{\text{bias}} &= \frac{J_{\text{FM}}}{m} \\ H_3 &= \frac{-2J_{\text{AFM}} + 2J_{\text{FM}} - \Delta E_3}{2m} \end{aligned} \tag{4}$$

where ΔE_1, ΔE_2, and ΔE_3 are the spin-flip barriers for each transition respectively. The strength of J_{FM} can be estimated by H_{E}.

Therefore, the main features in the hysteresis loops of $MnBi_4Te_7$ and $MnBi_6Te_{10}$ can be interpreted by the five-layer macrospin model. In the real material, there is inhomogeneity in the distribution of AFM and FM coupling, which might cause the discrepancy of switching ratio between experimental hysteresis loop and macrospin model.

Supplementary Note V. Cross-sectional STEM-EELS characterizations of $MnBi_4Te_7$ and $MnBi_6Te_{10}$ samples

The STEM images at the surface region show consistent stacking sequence (MBT+BT+BT) from inside bulk to the surface and no interlayer stacking disorders are observed. (Figs. S11a and b) Cross-sectional atomic-resolution high-angle annular dark-field scanning transmission electron microscopy (HAADF-STEM) image along the [100] direction of $MnBi_4Te_7$ shows an alternating structures of Bi_2Te_3 layers and $MnBi_2Te_4$ layers (Figs. S11c and d). The experimental and simulated integrated HAADF intensity profiles along the *c*-axis show clear discrepancy, showing Bi_{Mn} antisite defects in SL (red arrow in Fig. S11c) and Mn_{Te} in QL (grey arrow). In addition, atomic electron energy loss spectroscopy (EELS) mapping shows clear Mn signals in the Bi layers in SL and also QL (Fig. S11d), demonstrating the existence of the Mn_{Bi} antisite defects both in SL and QL.

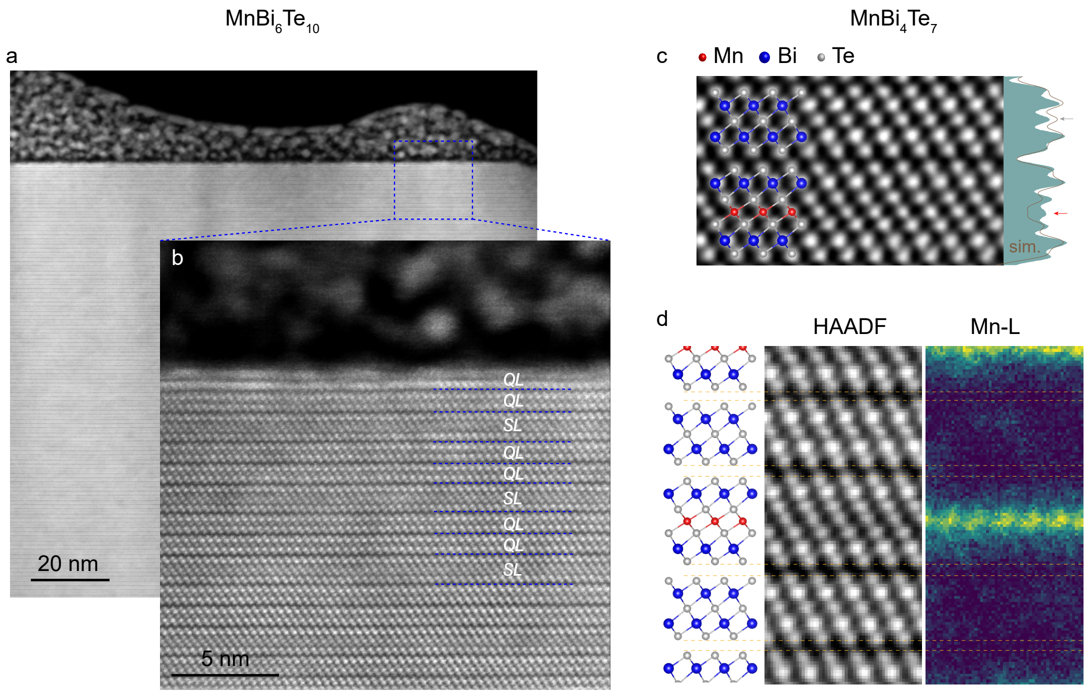

FIG. S11. **Cross-sectional STEM characterizations of $MnBi_6Te_{10}$ and $MnBi_4Te_7$ samples.** (a) Atomic-resolution HAADF image at the surface of $MnBi_6Te_{10}$ and (b) the enlarged image . We can see that the layers follow the $MnBi_6Te_{10}$ stacking sequence from inside bulk to the surface and no interlayer stacking disorders are observed. (c) Atomic-resolution HAADF image of the cross-section of the $MnBi_4Te_7$ crystal along the [100] direction. Experimental (shaded area) and simulated (grey curve) integral HAADF intensity profiles along the *c*-axis show clear discrepancy, showing Bi_{Mn} antisite defects in SL (red arrow) and Mn_{Te} in QL (grey arrow). (d) Atomic structure and HAADF image of the $MnBi_4Te_7$ crystal with the corresponding EEELs mapping of the Mn element ($L_{2,3}$ edge). Clear Mn signals are present in the Bi layers of SL and QL. The orange dashed lines indicate the van der Waals gaps.

Supplementary Note VI. RMCD measurements of few-layer $MnSb_2Te_4$ samples from three synthesized crystals

To gain insight of the possible effects of the ubiquitous defects on magnetism, we turn to explore the magnetism in $MnSb_2Te_4$ down to 1 SL. $MnSb_2Te_4$ is isostructural to $MnBi_2Te_4$, and these two compounds have the same types of defects. $MnSb_2Te_4$ crystals can be grown easily in a wide temperature range which makes it possible to tune the concentrations of lattice defects and hence the magnetism by varying the growth temperatures. As is expected, an evolution of A-type AFM to FM-AFM coexistence and finally to the FM ground state is observed with varying the Mn-Sb site-mixing concentration.

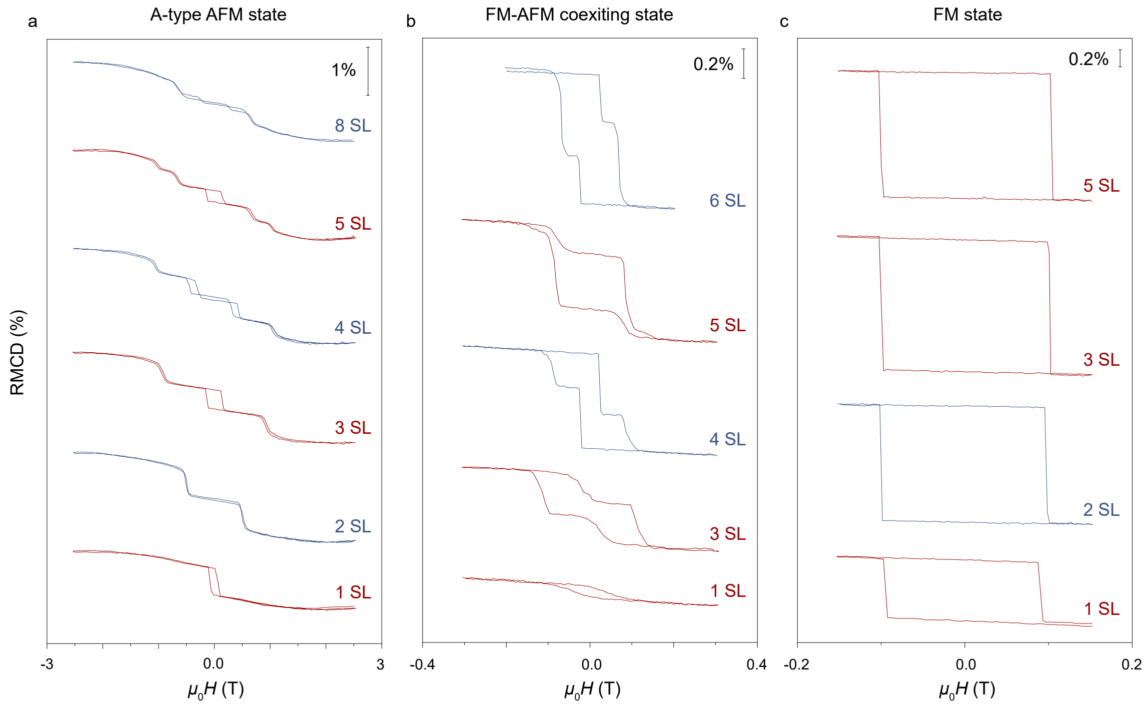

FIG. S12. **RMCD signals *versus* magnetic field measured at 2 K for layer-dependent $MnSb_2Te_4$ from three synthesized crystals.** The odd-layered and even-layered samples were plot in red and blue, respectively. (a) The $MnSb_2Te_4$-1 flakes show obvious odd-even layer-number effect, indicating an A-type AFM magnetic configuration. (b) The $MnSb_2Te_4$-2 flakes except 1 SL show multi-step spin-flip transitions with a decreased saturation field (< 0.2 T), indicating an FM-AFM coexisting magnetic configuration. (c) The $MnSb_2Te_4$-3 flakes show a rectangular magnetic hysteresis loop for all thicknesses, indicating a pure FM magnetic configuration.

Supplementary Note VII. Discussions of the FM-AFM domain distribution

To evaluate the domain sizes of the FM and AFM components, we also characterize the magnetic spatial homogeneity by RMCD mapping in $MnBi_4Te_7$. In a typical RMCD-$\mu_0 H$ curve of a thick $MnBi_4Te_7$ sample (Fig. S13a), we map the RMCD signals in a selected area (Fig. S13a, inset) under three selected magnetic fields (0.25 T, −0.1 T, and −0.25 T) corresponding to three different spin configurations (the plateaus around 0 T are hard to locate). The RMCD signals are uniform through the magnetic field sweep from state 1 to 3 (Figs. S13b-c) across the whole scanned area, indicating a homogeneous FM-AFM coexistence at a spatial resolution limited by the laser spot size of $\sim 2\ \mu$m.

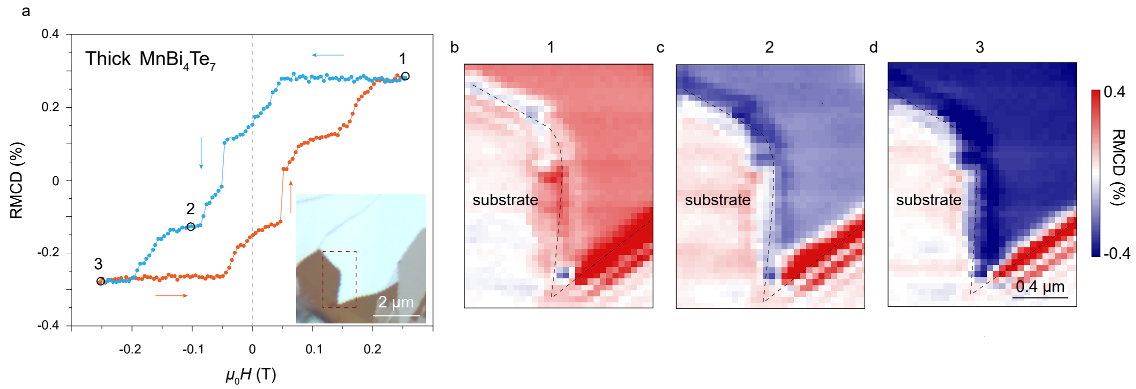

FIG. S13. **Spatial RMCD mappings of a thick $MnBi_4Te_7$ sample.** (a) RMCD signal *versus* external magnetic field of a thick $MnBi_4Te_7$ sample measured at 2 K. Three magnetic fields (0.25 T, −0.1 T, and −0.25 T) corresponding to three different spin configurations (labelled as 1 to 3) are selected for RMCD maping. Inset: optical image of the measured sample. (b-d) RMCD signal maps at 0.25 T, −0.1 T, and −0.25 T, respectively. The homogeneous signals indicate the uniform FM-AFM coexistence under the experimental spatial resolution.

It should be noted that these spin-flip transitions, especially for the FM spin-flip transition at H_c, are quite sharp despite the FM-AFM spatial inhomogeneity. In an inhomogeneous system, the sharp transition field suggests that its magnetic reversal is determined by the nucleation of reversed domain and the subsequent domain wall motion processes (i.e., the nucleation field is much higher than the propagation field)[7, 8]. We take the most representative 3-SL $MnBi_6Te_{10}$ as an example (Fig. S14a), we can see that the FM spin-flip transition (H_{c-}) is much sharper that the two AFM spin-flip transitions, and the second AFM spin-flip transition (H^2_{f-}) is sharper than the first AFM spin-flip transition (H^1_{f-}). To study these subtle differences, we need to consider the connectivity of the flipped components during these three spin-flip transitions. In the 3-SL sample, the two interlayer couplings would lead to four magnetic states (Fig. S14b), denoted by FF, FA, AF and AA (F for interlayer ferromagnetic coupling and A for interlayer antiferromagnetic coupling). To clarify the three spin-flip transitions discussed above and their connectivity, we performed a simplified simulation model that considered the spatial distributions of the four magnetic states in our sample.

We firstly generate a 200×200 matrix with randomly distributed FF, FA, AF, and AA magnetic states (each site represents a small uniform spin structure), as shown in Fig. S14c. Following the interlayer coupling and spin-flip rules under an external magnetic field, the spins in these four magnetic states will flip at H^1_{f-}, H_{c-} and H^2_{f-} as shown in Fig. S14d-g. We then focus on the detailed magnetic states at each transition. At H^1_{f-} where the AFM component flips, the flipped spins in these four magnetic states are highlighted with a grey background in Fig. S14h, where the FF region is not flipped and none of the other flipped regions are connected to each other. However, at H_{C-} where the FM component flips, except for the AA region which is not flipped, the other flipped regions are always connected to each other. Finally, at H^2_{f-} where the remaining AFM component flips, the AA region is always connected to FA and AF regions while the other regions cannot directly connect with each other.

Using these connectivity laws of the spin flips at H^1_{f-}, H_{C-} and H^2_{f-} (Fig. S14h), we can traverse the matrix in Fig. S14c and generate the connectivity matrices of the three spin-flip transitions, respectively. Specifically, at each spin-flip transition, for a magnetic state with coordinates (i, j) in the 200×200 matrix, if it has connectivity with the magnetic state at $(i-1, j)$ or $(i, j-1)$, it will be given the same value as the connected magnetic state, otherwise it will be given a new value (the specific value has no specific meaning). This assignment process will result in three new matrices with connected regions possessing the same values (color) and unconnected regions with new different values. Meanwhile, states that do not flip (such as FF state at the H^1_{f-} and H^2_{f-} transitions, and AA state at the

$H_{\mathrm{C}-}$ transition) are shown in white. In this way, we can obtain a connectivity map for each spin-flip transition (Fig. S14i-k), where colored regions represent flipped states and white regions represent states that do not involve in that transition.

We can see that the connectivity map at $H_{\mathrm{f}-}^1$ shows a random discontinuous distribution (Fig. S14i), while the connectivity map at $H_{\mathrm{C}-}$ shows roughly a single color of connectivity (Fig. S14j). Because of its excellent connectivity, for spin-flip transition of the FM component at $H_{\mathrm{C}-}$, even though the coupling strengths of each region are not exactly the same, once the reverse domain is nucleated, the DW will propagate across the sample to flip most of the FM component. This results in a sharp transition during the FM component reversal, which is in good agreement with what we observed experimentally. For spin-flip of the AFM component at $H_{\mathrm{f}-}^1$ (Fig. S14i), due to the disconnectivity and discrete distributions of the individual flipped regions, the process of nucleation and DW propagation will occur successively in each small area because of the inhomogeneous AFM interlayer coupling strength, resulting in a smeared transition (with a broad distribution of spin-flip field) compared with the $H_{\mathrm{C}-}$ for FM component. We should also note that from the RMCD results (Fig. S14a), the transition at $H_{\mathrm{f}-}^2$ is sharper than that at $H_{\mathrm{f}-}^1$, but still more sluggish than that at $H_{\mathrm{C}-}$, which can also be explained by the corresponding connectivity. The connectivity map at $H_{\mathrm{f}-}^2$ shows that its flipped regions are generally connected, but there are still some isolated island-liked connected regions scattered (Fig. S14k). Given the agreement between the experimental observations and the simulation results from our model, we believe that the magnetic reversal is determined by the nucleation of reversed domain and the subsequent DW motion processes, which indeed requires connectivity between flipped magnetic states for the continuous motion of the DW.

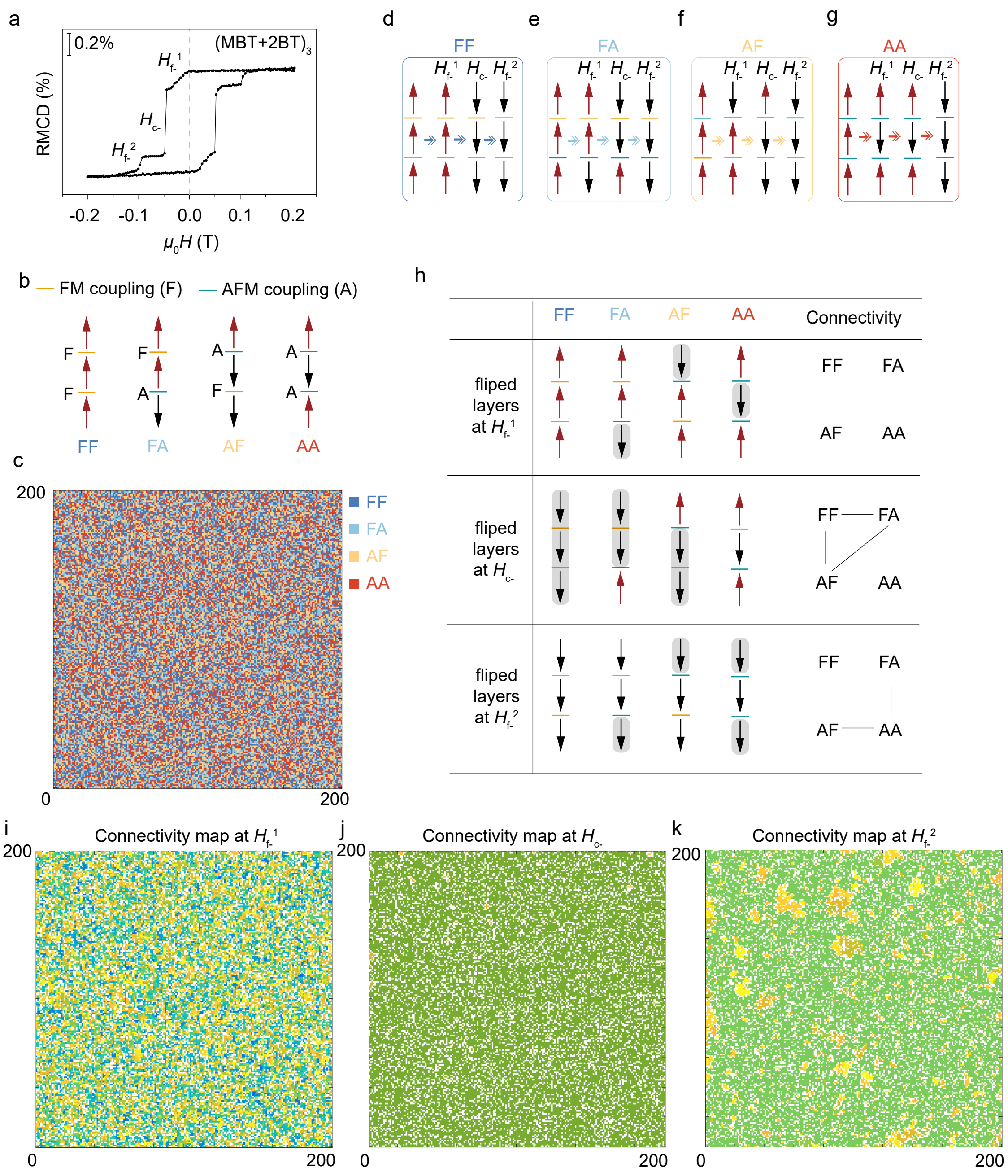

FIG. S14. **Magnetic reversal model of a 3-SL $MnBi_6Te_{10}$ sample.** (a) RMCD signal *versus* external magnetic field of a 3-SL $MnBi_6Te_{10}$ flake at 2 K. (b) Four magnetic states considered in the 3-SL sample. (c) Simulation of the spatial distributions of the four magnetic states. (d-g) Schematic diagrams of the spin flips of the four magnetic states at H^1_{f-}, H_{C-} and H^2_{f-}, respectively, as the magnetic field descends. (h) Connectivity analysis at each spin-flip transition. (i-k) Connectivity maps at H^1_{f-}, H_{C-} and H^2_{f-}, respectively.

Supplementary Note VIII. Single-crystal X-ray diffraction characterizations of $MnBi_4Te_7$ and $MnBi_6Te_{10}$

The crystal structure of the as-grown $MnBi_4Te_7$ (Table. I) and $MnBi_6Te_{10}$ (Table. II) crystals were elucidated via single-crystal X-ray diffraction (SCXRD) measurement. Since the cation antisite defects are ubiquitous in MBT-type compounds[9–11], antisite mixing of Bi and Mn was taken into account in the structural refinement by allowing Bi to occupy Mn site and vice versa. The results indicate that with the intercalated Bi_2Te_3 layers, the Mn_{Bi} antisites increase from 0.5 % to 3 % and the Bi_{Mn} antisites also increase from 26 % to 49 % from $MnBi_4Te_7$ to $MnBi_6Te_{10}$. Combing with the STEM results, we can conclude the prevalent antisite defects distributed through the whole crystals.

TABLE I. **Crystallographic data for $Mn_{0.75}Bi_{4.25}Te_7$, refined with Bi antisite defect from an SCXRD experiment.**

Space group	$P\bar{3}m1$ (No. 164)
Lattice parameters (Å)	a = b = 4.3788, c = 23.8536
	$\alpha = 90°, \beta = 90°, \gamma = 120°$
Cell volume (Å^3)	396.08
Density (g cm^{-3})	7.641
Structure parameters:	

Atom	Site	Symmetry	x	y	z	Occup.	U_{eq}
Bi1	2d	3m	0.66667	0.33333	0.08583	0.995	0.018
Mn1	2d	3m	0.66667	0.33333	0.08583	0.005	0.018
Bi2	2d	3m	-0.33333	1.33333	0.34173	1.000	0.018
Te1	1a	-3m	1.00000	0.00000	0.00000	1.000	0.014
Te2	2d	3m	0.33333	0.66667	0.15872	1.000	0.015
Te3	2c	3m	0.00000	1.00000	0.27015	1.000	0.013
Te4	2d	3m	-0.66667	-1.66667	0.43160	1.000	0.016
Mn3	1b	-3m	-1.00000	2.00000	0.50000	0.740	0.019
Bi1	1b	-3m	-1.00000	2.00000	0.50000	0.260	0.019

TABLE II. **Crystallographic data for $Mn_{0.67}Bi_{6.33}Te_{10}$, refined with Bi antisite defect from an SCXRD experiment.**

Space group	$R\bar{3}m$ (No. 166)
Lattice parameters (Å)	a = b = 4.3867, c = 102.24
	$\alpha = 90°, \beta = 90°, \gamma = 120°$
Cell volume (Å^3)	1703.8
Density (g cm^{-3})	7.705
Structure parameters:	

Atom	Site	Symmetry	x	y	z	Occup.	U_{eq}
Mn1	3a	-3m	0.00000	0.00000	0.00000	0.510	0.134
Bi1	3a	-3m	0.00000	0.00000	0.00000	0.490	0.134
Te1	6c	3m	-0.33333	-0.33333	-0.01662	1.000	0.051
Bi2	6c	3m	-0.66667	0.66667	-0.03706	0.970	0.039
Mn2	6c	3m	-0.66667	0.66667	-0.03706	0.030	0.039
Te2	6c	3m	-1.00000	1.00000	-0.05458	1.000	0.045
Bi3	6c	3m	-1.66667	1.66667	-0.09665	1.000	0.022
Te3	6c	3m	-1.33333	1.33333	-0.08014	1.000	0.022
Bi4	6c	3m	-2.33333	2.33333	-0.13628	1.000	0.029
Te4	6c	3m	-2.00000	2.00000	-0.11682	1.000	0.039
Te5	6c	3m	-2.66667	2.66667	-0.15357	1.000	0.051

Supplementary Note IX. The exchange bias effect of the FM component

To explore the stability of the exchange bias effect of the FM component in MBT systems, here, we take $MnBi_6Te_{10}$ for an example. The large-field full hysteresis loop (grey data in Fig. S15) is plotted as reference for the minor hysteresis loops of the FM components. Historically polarized by a large positive saturation magnetic field, the minor hysteresis loop of the FM component shifts to the left side as we discussed in the main text. For magnetic field sweeping back and forth four times, all the minor loops overlap each other, showing no training effect and confirming the stability of the coupling between the AFM and FM components.

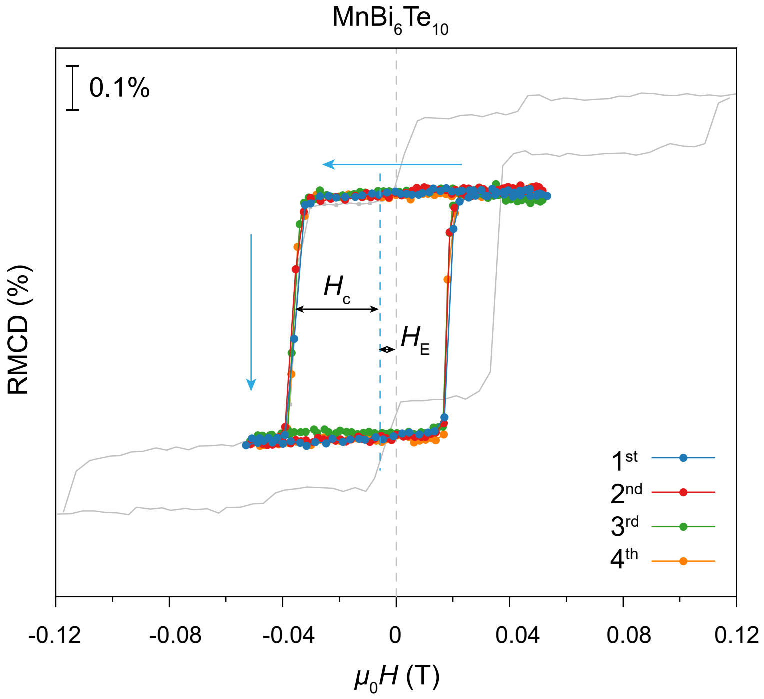

FIG. S15. **The stability of the exchange bias effect of the FM component in a $MnBi_6Te_{10}$ sample.** The large-field full hysteresis loop is plotted as reference with grey color. The hysteresis loop of the FM component is measured by back-and-forth magnetic field sweeps for four times with different colors. The exchange bias is very stable, and no training effect is observed.

[1] Xie, H. *et al.* The mechanism exploration for zero-field ferromagnetism in intrinsic topological insulator $MnBi_2Te_4$ by Bi_2Te_3 intercalations. *Appl. Phys. Lett.* **116**, 221902 (2020).
[2] Klimovskikh, I. I. *et al.* Tunable 3d/2d magnetism in the $(MnBi_2Te_4)(Bi_2Te_3)_m$ topological insulators family. *npj Quantum Mater.* **5**, 1–9 (2020).
[3] Stoner, E. C. & Wohlfarth, E. A mechanism of magnetic hysteresis in heterogeneous alloys. *Philos. Trans. Royal Soc. A* **240**, 599–642 (1948).
[4] Yang, S. *et al.* Odd-even layer-number effect and layer-dependent magnetic phase diagrams in $MnBi_2Te_4$. *Phys. Rev. X* **11**, 011003 (2021).
[5] Yan, J.-Q. *et al.* A-type antiferromagnetic order in $MnBi_4Te_7$ and $MnBi_6Te_{10}$ single crystals. *Phys. Rev, Mater.* **4**, 054202 (2020).
[6] Lai, Y., Ke, L., Yan, J., McDonald, R. D. & McQueeney, R. J. Defect-driven ferrimagnetism and hidden magnetization in $MnBi_2Te_4$. *Phys. Rev. B* **103**, 184429 (2021).
[7] Franken, J. H., Hoeijmakers, M., Lavrijsen, R. & Swagten, H. J. M. Domain-wall pinning by local control of anisotropy in pt/co/pt strips. *Journal of Physics: Condensed Matter* **24**, 024216 (2011).
[8] Cullity, B. D. & Graham, C. D. *Domains and the Magnetization Process*, chap. 9, 275–333 (John Wiley Sons, Ltd, 2008).
[9] Liu, Y. *et al.* Site mixing for engineering magnetic topological insulators. *Phys. Rev. X* **11**, 021033 (2021).
[10] Vidal, R. C. *et al.* Topological electronic structure and intrinsic magnetization in $MnBi_4Te_7$: a Bi_2Te_3 derivative with a periodic Mn sublattice. *Phys. Rev. X* **9**, 041065 (2019).
[11] Souchay, D. *et al.* Layered manganese bismuth tellurides with $GeBi_4Te_7$-and $GeBi_6Te_{10}$-type structures: towards multifunctional materials. *J. Mater. Chem. C* **7**, 9939–9953 (2019).
